# The live-streaming strategy for competitive manufacturers considering information disclosure and product heterogeneity

**DOI:** 10.1371/journal.pone.0339997

**Published:** 2026-02-11

**Authors:** Liwen Liu, Qing Wang, Zonghuo Li, Tingting Wu, Yuan Yuan

**Affiliations:** 1 School of Politics and Public Administration, Soochow University, Wuzhong District, Suzhou, Jiangsu, China; 2 School of Business, Zhengzhou University of Technology, Huiji District, Zhengzhou, Henan, China; MCC Boyd Tandon School of Business, INDIA

## Abstract

Live-streaming has become increasingly populous in recent years and a large body of manufacturers such as Apple and Huawei have adopted live-streaming strategies. Despite the live-streaming can disclose product information and enhance consumer utilities, consumers may leave the market if they realize that the product is not fit for them after watching the live-streaming. This paper studies the opening strategy of live-streaming for competitive manufacturers who sell products with differential quality. Four cases regarding whether the manufacturers open a live-streaming channel to disclose product information are modeled, namely, NN (no manufacturer opens), DN/ND (one manufacturer opens), and DD (both manufacturers open). We further analyze how product quality influences live-streaming strategies. The result shows that adopting live-streaming allows a manufacturer to charge higher prices, whereas competitor live-streaming adoption exerts downward price pressure. Interestingly, live-streaming does not universally benefit manufacturers. Rather, for the high-quality manufacturer, the manufacturer benefits from the live-streaming channel when the fitness of its product is mid-value. However, for the low-quality manufacturer, the manufacturer will be better off by opening a live-streaming channel when the fitness of its product is large.

## 1. Introduction

Live streaming retail, a new sales model based on real-time video, has become an indispensable marketing tool for businesses in recent years and has expanded rapidly worldwide. For example, during China’s 2022 “Double 11” shopping festival, live streaming sales reached $25.7 billion, representing a year-on-year growth of 146.1% [1]. As of 2021, there were 76,000 live e-commerce enterprises in China—a increase of 216.66% compared to the previous year—with a user base of 620 million and a total transaction value exceeding RMB 5.3 trillion [2]. International e-commerce platforms such as Facebook, Amazon, and Walmart have also introduced live streaming features through partnerships with platforms like Instagram and TikTok, further demonstrating the model’s commercial value.

Unlike traditional online sales methods, live-streaming sales involve hosts introducing products to consumers via real-time video on merchant platforms [3]. The format and content of live streams—such as interactive games between hosts and viewers, the authenticity of product experience sharing, and trust-assurance cues embedded in the live-streaming interface—all significantly influence consumer engagement and purchasing behavior. During broadcasts, hosts present products from multiple perspectives using images, videos, and textual descriptions [4]. This allows viewers to acquire more detailed product information through demonstrations [5], pose questions freely in the comment section, and engage in discussions with others [6], thereby developing a more comprehensive understanding of the products [7]. Grounded in theories such as customer participation and uses and gratifications, live streaming fulfills diverse consumer needs by offering informational, entertainment, and social experiences. The real-time interaction between hosts and consumers mitigates the lack of social presence typical in conventional online shopping [8], reduces uncertainty in product evaluation [9], and diminishes perceived risks associated with online purchases [10]. As a result, it strengthens trust and purchase intention [11,12], while also enhancing participation and driving purchasing behavior [13].

The reasons enterprises adopt live-streaming channels are diverse, including highlighting product authenticity and advantages, building trust, deepening interaction, leveraging platform incentives, and balancing operational costs against returns. Based on the theories discussed above, companies can formulate corresponding strategies. These may include adopting live-streaming channels to enhance the quality of host-viewer interaction and strengthen the sense of social presence; incorporating authentic and entertaining content to meet user needs; designing highly interactive live-stream segments to foster customer integration; and optimizing trust mechanisms and process flows—all of which contribute to higher conversion rates and stronger consumer trust. For example, Nongfu Spring conducted live streams that took viewers on a virtual “factory tour,” showcasing its water sources, modern production lines, and rigorous quality inspection processes in real time. This approach intuitively emphasized the brand’s core values of “natural and healthy” products, effectively addressing consumer concerns about water safety.

In the live streaming ecosystem, manufacturer self-streaming—exemplified by brands such as Apple, Huawei, and Suning—has emerged as one of the mainstream formats. In 2020, approximately 55% of live streams on Taobao were conducted by sellers themselves (iResearch, 2021), underscoring the growing significance of this trend. In such broadcasts, manufacturers independently set prices and sell their own products [13], thereby creating brand-exclusive live streaming environments [14]. However, as more manufacturers venture into live streaming—for instance, both Apple and Huawei launched their live streaming channels in 2023—competition has intensified. This expansion is often accompanied by high operational costs and the potential for price wars.

Notably, current research in live streaming has predominantly centered on consumer trust [15,16,17], platform or channel mechanisms [18,19,20], and the influence of influencers [21,22,23]. However, comparatively less attention has been devoted to the competitive strategies of heterogeneous manufacturers in live streaming under conditions of quality differences. In practice, competitive manufacturers may sell heterogeneous products with distinct quality [24] and product fitness is private information. Consumers do not acknowledge the product information in advance and purchase a suitable product based on their utility perception. Therefore, live-streaming provides consumers with detailed product information and thus, consumers obtain higher utility if products fit with their expectations [25]. Nonetheless, it is uneconomic for manufacturers to adopt live-streaming if the product is not suitable for consumers since consumers will leave the market and the manufacturers undertake an operation cost for live-streaming. Therefore, whether it is beneficial for manufacturers, especially low-quality manufacturers, to disclose product information to expand market sizes is unknown. How to make a live-streaming strategy based on product quality, consumer preference, and the live-streaming strategy of competitors is far from resolved. It is unclear how these factors affect the manufacturers’ live-streaming choices.

Although live-streaming sales have experienced rapid practical growth, the theoretical underpinnings and strategic implications outlined above have not yet been systematically investigated. In particular, there is a lack of modeling and analytical research from a game-theoretic perspective regarding manufacturers’ decisions to adopt live streaming and their quality information disclosure mechanisms. To bridge this research gap, this study develops a game-theoretic model involving two competing manufacturers, incorporating product quality differences and consumer preference heterogeneity. The aim is to provide a theoretical foundation and practical decision support for manufacturers formulating strategies in the context of live-streaming commerce. Four cases regarding whether the competitive manufacturers adopt live-streaming channels are modeled, including NN (no manufacturer opens), DN/ND (one manufacturer opens), and DD (both manufacturers open). We further explore the impact of product quality on the opening strategy of live-streaming. Consumers hold an expected utility in the absence of live-streaming due to information asymmetry, whereas they could realize full utility in the presence of live-streaming. The optimal pricing strategy and opening strategy of live-streaming channel are revealed.

This paper contributes theoretically by shedding light on the operational mechanism of live-streaming for competitive manufacturers when manufacturers sell quality differential products. Practically, we provide a pricing strategy and market-capturing policy for manufacturers considering the live-streaming of the competitor and consumer matching. Furthermore, we suggest a decision-making system for manufacturers to clearly understand the conditions of adopting live-streaming selling as a sales channel.

The remainder of this paper is arranged as follows. In Section 2, we review the relative literature. In Section 3, we describe the model structure and decision problem. This paper sets up the model in Section 4. Model analysis are investigated in Section 5. Section 6 concludes the results and points out some directions for further research. All the proofs are provided in the Supporting Information.

## 2. Literature review

The literature review in this article addresses two primary research streams: live-streaming selling and consumer preferences. It seeks to bridge them theoretically and provide a foundation for the proposed study. The following section organizes the literature around thematic categories, clarifies the theoretical positioning of this research, and identifies the specific gaps it aims to fill.

### 2.1 Live streaming selling

Live-streaming selling, as an emerging sales model, can be deeply explained through several theoretical frameworks: signaling theory, information asymmetry theory, and consumer engagement theory. According to signaling theory, live-streaming serves as a credible signal of product quality. In markets characterized by information asymmetry, high-quality manufacturers can make substantial investments in live-streaming—such as hiring professional hosts, offering multi-angle real-time demonstrations, and providing unconditional return policies—to convey confidence in their products [26,7]. These signals are credible because they are difficult for low-quality manufacturers to imitate. Second, live-streaming significantly reduces uncertainty resulting from information asymmetry. By enabling real-time video displays, interactive Q&A with hosts, and public discussions in comment sections, implicit product attributes—such as actual size, texture, and user experience—are transformed into explicit, verifiable information [8]. This process enriches consumers’ information sets, lowers uncertainty in utility evaluation, and thus enhances purchase intention. Finally, consumer engagement theory offers a framework for understanding how live-streaming builds interaction and trust. Unlike one-way communication, live-streaming creates a highly interactive and immersive environment [6]. Hosts engage the audience through quizzes, giveaways, and limited-time promotions, stimulating cognitive and emotional involvement [27]. Viewers become active participants and co-creators of content rather than passive recipients. This deep level of engagement not fulfills social and entertainment needs—as explained by the uses and gratifications theory [28]—but also fosters emotional connection and trust between consumers and brands or hosts [11]. Consequently, it strengthens brand loyalty and facilitates long-term customer relationships.

Existing research on live streaming selling can be categorized into three main themes: First, consumer behavior and participation mechanisms. Scholars have primarily explained how live streaming stimulates purchase intention through trust-building [16,17], cognitive-affective dual pathways [15], and social presence [8]. For example, Sun et al. [10], from a technological affordance perspective, argued that live streaming enhances consumer participation intention by improving interactivity and authenticity. Gu et al. [22] identified an inverted U-shaped relationship between entertainment intensity and participation, suggesting that content strategies should be moderated appropriately. While these studies emphasize user experience, they often fail to fully incorporate consumer engagement theory or systematically analyze how live streaming serves as a signaling mechanism to reduce quality uncertainty. Second, manufacturers’ live streaming adoption strategies (also related to platform or channel mechanisms). Research indicates that manufacturers’ decisions to adopt live streaming depend on factors such as sales capabilities, commission structures, operational costs, and product attributes [18,29,30,31]. Li et al. [19] further noted that live streaming is more suitable for products with low price sensitivity, as it more effectively communicates non-price value. However, these studies predominantly focus on operational efficiency and cost-effectiveness, lacking discussion on quality disclosure strategies within an information asymmetry framework. They also do not treat the choice of live streaming as a strategic signaling game in competitive contexts. Third, the influencer effect (including platform governance and host contract design). Recent studies have begun examining contractual structures between platforms and hosts [32,23] and how platform governance influences host behavior [20]. This body of work offers an institutional perspective on the live streaming ecosystem, yet it does not extend to critical questions regarding how manufacturers can use live streaming to signal product suitability.

### 2.2 Consumer preference

According to consumer decision-making theory, preferences refer to consumers’ relatively stable evaluations and rankings of different product attributes—such as quality, brand, functionality, and appearance [33]. These preferences form a mental benchmark for an “ideal product.” Among these, quality preference is particularly crucial, as it determines consumers’ willingness to pay a premium for higher quality [34]. For example, some consumers prioritize high performance (reflecting a high-quality preference), while others prefer basic functionality (reflecting a lower quality preference). Research on consumer preferences often focuses on how quality preferences influence firms’ strategies [35,5 21,36,37]. For instance, examined a differentiated duopoly game and explored how varying degrees of consumer preference dispersion affect decision-making. Zhang and Li [37] demonstrated that firms can implement behavior-based price discrimination by strategically disclosing quality information to different customer segments. However, most studies in this stream of literature assume that consumers are already informed about product quality before purchase, overlooking contexts in which actual product fit constitutes private information.

In online environments, the actual suitability of products—such as how well clothes fit, whether a mobile phone’s performance meets expectations, or if a skincare product matches one’s skin type—constitutes hidden information for consumers, leading to significant information asymmetry [38]. This uncertainty generates perceived risk [39], wherein consumers fear that products may not meet their needs, potentially resulting in financial or psychological losses. The volume and quality of product information serve as key mechanisms to mitigate this uncertainty. Drawing on information processing theory, consumers actively seek information to bridge “knowledge gaps” and make more accurate judgments [40]. Live streaming addresses this by delivering a rich array of multi-dimensional information—such as dynamic demonstrations, real-time Q&A, and user comments—going far beyond traditional text and images. Although several studies have begun to examine how information volume influences suitability judgments [41, 42] and have explored corporate adaptability disclosure strategies [43,44,45,46,47], these issues remain underexplored in the context of live streaming. For instance, while Jia [48] and Xie et al. [49] emphasized the role of balanced disclosure and virtual try-ons in building trust and reducing return rates, their studies did not fully theorize the underlying mechanisms nor investigate strategic interactions within a competitive multi-manufacturer environment.

In summary, the current research exhibits the following core gaps: First, studies on live-streaming sales lack deep integration with foundational theories such as signaling theory, information economics, and consumer engagement theory. As a result, they fail to systematically explain how live-streaming mitigates quality uncertainty and adaptation risks through real-time interaction and multi-dimensional information disclosure. Second, although consumer preference research is well-established, its intersection with live-streaming remains underexplored. There is a particular scarcity of analysis from a competitive game-theoretic perspective regarding how manufacturers can utilize live-streaming to disclose information about both product quality and fit.

To address the aforementioned research gaps, this study constructs a duopoly model involving two manufacturers with differentiated product quality, where information about product fit is private. Grounded in information asymmetry and signaling theory [26], we conceptualize live streaming as an effective mechanism for manufacturers to convey credible signals. Drawing on consumer utility theory, the model incorporates a utility function that accounts for product quality, perceived fit, and the informational value provided through live streaming. Furthermore, by integrating insights from the Hotelling model and Bertrand competition logic, we analyze firms’ disclosure strategies and investment in live-streaming capabilities under quality differentiation. This study not only bridges the theoretical disconnect between live streaming and adaptation preference but also offers practical decision support for manufacturers aiming to devise disclosure and competitive strategies in live-streaming environments based on their product quality. Table 1 presents a comparative summary of existing literature and this study to further elucidate its research positioning and theoretical contributions (LS indicates live-streaming).

**Table 1:** Summary of related literature.

	LS	LS selling decision	Consumer quality preferences	Product fit
Gal-Or et al. [41]				✓
Gu et al. [43]				✓
Branco et al. [50]				✓
Cui et al. [34]			✓	✓
Sun et al. [10]	✓			
Han et al. [35]			✓	
Sun et al. [44]				✓
Xu et al. [15]	✓			
Shi [45]				✓
Sun et al. [5]			✓	
Wu et al. [46]				✓
Chen et al. [16]	✓			
Alventosa et al. [51]			✓	
Du et al. [18]	✓	✓		
Zhang et al. [3]	✓	✓		
Li et al. [19]	✓	✓		
Gu et al. [22]	✓			
Zhang et al. [42]	✓	✓		
Liu et al. [21]			✓	
Jia [48]	✓			✓
Xie et al. [49]				✓
Hong et al. [36]			✓	
Zhang et al. [37]			✓	
Pu et al. [17]	✓			
This paper	✓	✓	✓	✓

## 3. Problem description and model setting

This study develops a game-theoretic model involving two competing manufacturers ($M_{\text{1}}$ and $M_{\text{2}}$) that incorporates quality differences and consumer preference heterogeneity, aims to answer the following research questions: (1) Under what conditions can live streaming enhance manufacturers’ profits? (2) How does competition affect manufacturers’ live-streaming strategies? (3) What role does product quality play in the effectiveness of live streaming? By addressing these questions, this research provides theoretical insights and practical guidance to help manufacturers design effective live-streaming strategies in competitive markets.

Specifically, $M_{\text{1}}$ produces products with a higher quality, denoted by $z_{\text{1}}=\text{1}$. While the product quality of $M_{\text{2}}$ is $z_{\text{2}}=\delta$, where 0<δ≤1, indicating a lower quality level. Both manufacturers sell products through direct selling channel with price $p_{\text{1}}\;$ and $\;\;p_{\text{2}}$, respectively, and incur production costs $c_{\text{1}}$ and $c_{\text{2}}$. The prices and costs satisfy the conditions: $p_{\text{1}}>c_{\text{1}}>\text{0}\;and\;p_{\text{2}}>c_{\text{2}}>\text{0}$.

The manufacturers’ products exhibit both vertical and horizontal attributes [52,53]. Vertical differentiation is captured through product quality, which is assumed to be known to consumers. Horizontal differentiation reflects non-quality characteristics—such as performance or taste—which constitute private information prior to purchase, leading to information asymmetry [38]. The market size is normalized to 1. Consumers are heterogeneous in their fit preferences with the products: among the market, a fraction of α(0≤α≤1) consumers are suitable for the product of $M_{\text{1}}$; a fraction of β(0≤β≤1) consumers are suitable for the product of $M_{\text{2}}$; a fraction of αβ consumers are suitable for the product of $M_{\text{1}}$ and $M_{\text{2}}$ simultaneously; a fraction of (1−α−β+αβ) consumers are unsuitable for any product and quit the market. We denote α and β the fitness of $M_{\text{1}}$’s and $M_{\text{2}}$’s products, respectively. Consumers receive additional utility $x_{i},\text{0}<x_{i}<\text{1},(i=\text{1},\text{2})$ if the product is suitable for them. On the contrary, consumers will not buy the product if unsuitable.

Each manufacturer decides whether to adopt a live-streaming channel to disclose product information. Live streaming serves as a signaling mechanism [26]: if a manufacturer chooses to stream live, it conveys credible information through real-time interaction and multi-dimensional display, transforming unobservable horizontal attributes into visible information. This enables consumers to accurately assess product utility. If a manufacturer does not live-stream, consumers can only form expected utility based on observable quality signals ($z_{i}$), yet remain uncertain about product fit. Adopting live streaming requires a fixed cost $C_{di}>\text{0}$, i=1,2, which is independent of sales volume and covers basic inputs such as equipment and hosting fees.

Based on the manufacturers’ binary decision of whether to adopt live streaming, this study examines four scenarios: (i) neither manufacturer conducts live streaming (Case NN); (ii) only $M_{\text{1}}$ conducts live streaming (Case DN); (iii) only $M_{\text{2}}$ conducts live streaming (Case ND); and (iv) both manufacturers conduct live streaming (Case DD).

To construct a clear and analytically tractable theoretical model, each assumption is grounded in established theoretical frameworks and abstracts from real-world complexity within reasonable bounds, so as to focus on the core mechanisms of manufacturer competition:

Assumption 1 (Duopoly Market Structure): While real markets involve multiple brands, platforms, and influencers, this study focuses on the core competitive interaction between two manufacturers. This is consistent with the classical oligopoly framework in industrial organization theory [54]. A duopoly model allows us to capture essential strategic interactions—such as price and quality competition—while maintaining analytical tractability. This approach is well-established in studies examining quality differentiation and information disclosure strategies (e.g., [55]), thereby providing a solid theoretical foundation for the current model.

Assumption 2 (Product Quality and Consumer Perception): We assume that manufacturer $M_{\text{1}}$ offers a higher-quality product than $M_{\text{2}}$ (i.e., $z_{\text{1}}>z_{\text{2}}$), grounded in the theory of vertical differentiation [56]. Furthermore, consumers are assumed to have complete information regarding product quality. This assumption is reasonable in the context of mature product categories where consumers have established brand awareness and perception [5].

Assumption 3 (Consumer Behavior): Consumers are modeled as rational utility-maximizing agents. This assumption is derived from rational choice theory in microeconomics [54] and provides a clear benchmark for analyzing trade-offs among price, quality, and product fit. This simplification allows us to isolate the core competitive mechanisms in live-streaming strategies [57].

Assumption 4 (Informational Role of Live Streaming): In this study, live streaming is modeled as a complete and truthful information disclosure mechanism, abstracting from implementation-related variations such as content quality and host credibility. This allows us to focus on the strategic incentives for firms to disclose information. The theoretical basis for this approach stems from signaling theory in information economics [26], which suggests that live streaming can serve as a high-quality credible mechanism for manufacturers to convey signals about product quality and fit.

Assumption 5 (Audience Targeting): The model presented in this thesis assumes that live streaming reaches the target audience—that is, consumers suited to a particular product will watch the corresponding manufacturer’s live stream. This approach allows us to focus on the impact of manufacturers’ live-streaming information disclosure within a controlled strategic environment. This aligns with existing literature on information targeting (e.g., [58]).

Assumption 6 (Cost Structure): The cost of live streaming, denoted as $C_{di}$, is modeled as a fixed cost that is independent of audience size or sales revenue. This approach is well-established in the literature analyzing technology and strategy adoption decisions (e.g., [59]), enabling the analysis to focus on the strategic value of live streaming rather than the specifics of its cost structure.

Assumption 7 (Static Competition): This study employs a static game framework in which firms make simultaneous one-time decisions. This approach is standard in game-theoretic analyses of competitive strategy, as it facilitates the derivation of clear equilibrium outcomes and enhances the understanding of strategic interactions among firms [60], thereby providing a foundation for assessing the strategic value of live streaming.

Assumption 8 (Demand Modeling): In this study, consumer demand is modeled in the form of market share (choice probability) rather than absolute sales volume. This approach is grounded in the theoretical of discrete choice models [61] and is particularly suitable for analyzing firms’ relative attractiveness and competition for market share in a competitive setting. The current model focuses on such relative demand—expressed as probability shares—to examine how changes in market size, such as product fit probability, affect manufacturers’ profitability in live streaming.

## 4. The model

### 4.1 No one adopts live-streaming (Case NN)

In the scenario where neither manufacturer opens a live-streaming channel, consumers cannot ascertain whether a product fits their preferences, resulting in decision-making under imperfect information. This reflects a typical setting of information asymmetry [38], where consumers form expected utility based on probabilistic assessments of product suitability. According to the theory of consumer utility, the utility consumers acquired for $M_{\text{1}}$ and $M_{\text{2}}$ are $U_{\text{1}}=\alpha (\theta z_{\text{1}}+x_{\text{1}})-p_{\text{1}}$ and $U_{\text{2}}=\beta (\theta z_{\text{2}}+x_{\text{2}})-p_{\text{2}}$, respectively. θ is the valuation consumers perceive from the product and follows a uniform distribution over [0,1]. Consumers buy products depending on max{*U*_*1*_, *U*_*2*_, 0}. Then we have $U_{\text{1}}>U_{\text{2}}>\text{0}$ when $\frac{\alpha (p_{\text{2}}-\beta x_{\text{2}})}{\beta (p_{\text{1}}-\alpha x_{\text{1}})}<\delta <\text{1}$. Accordingly, the market demands for the two products are as follows:


$$D_{\text{1}}=\frac{\alpha -\beta \delta +\alpha x_{\text{1}}-\beta x_{\text{2}}-p_{\text{1}}+p_{\text{2}}}{\alpha -\beta \delta }$$ (1)

$$D_{\text{2}}=\frac{-\alpha \beta \delta x_{\text{1}}+\alpha \beta x_{\text{2}}+\beta \delta p_{\text{1}}-\alpha p_{\text{2}}}{\beta \delta (\alpha -\beta \delta )}$$ (2)

The two manufacturers engage in Bertrand competition with differentiated products, each aiming to maximize profit:


$$\underset{p_{\text{1}}}{Max}\;\;\pi_{M_{\text{1}}}^{NN}=(p_{\text{1}}-c_{\text{1}})D_{\text{1}}=(p_{\text{1}}-c_{\text{1}})\frac{\alpha -\beta \delta +\alpha x_{\text{1}}-\beta x_{\text{2}}-p_{\text{1}}+p_{\text{2}}}{\alpha -\beta \delta }$$ (3)

$$\underset{p_{\text{2}}}{Max}\;\;\pi_{M_{\text{2}}}^{NN}=(p_{\text{2}}-c_{\text{2}})D_{\text{2}}=(p_{\text{2}}-c_{\text{2}})\frac{-\alpha \beta \delta x_{\text{1}}+\alpha \beta x_{\text{2}}+\beta \delta p_{\text{1}}-\alpha p_{\text{2}}}{\beta \delta (\alpha -\beta \delta )}$$ (4)

Driving the equilibrium solutions allows us to have the following results.

**Property 1.** *When no one adopts live-streaming,*

(1) $\pi_{M_{\text{1}}}^{NN}$ *is a concave function with respect to* $p_{\text{1}}$*, indicating there is maximum value for* $\pi_{M_{\text{1}}}^{NN}$.
(2) $\pi_{M_{\text{2}}}^{NN}$ *is a concave function with respect to* $p_{\text{2}}$*, indicating there is maximum value for* $\pi_{M_{\text{2}}}^{NN}$.

Property 1 shows that there exist optimal pricing strategies $p_{\text{1}}$ and $p_{\text{2}}$ such that both manufacturers receive maximum profit in product competition.

**Proposition 1.** *In Case NN, the equilibrium prices, market demands, and profits can be acquired with the conditions* $\alpha >\beta \delta >c_{\text{1}}$*,* *, and*

$\frac{\text{2}\alpha (\alpha -\beta \delta )-c_{\text{1}}(\text{2}\alpha -\beta \delta )+\alpha c_{\text{2}}}{\alpha \beta }<x_{\text{2}}<\frac{\beta \beta \delta \delta +c_{\text{2}}(\text{2}\alpha -\beta \delta )-\beta \delta c_{\text{1}}}{\beta (\text{2}\alpha -\beta \delta )}$ *. The equilibrium solutions are*


$$p_{\text{1}}^{NN*}=\frac{\text{2}\alpha (\alpha -\beta \delta )+\text{2}\alpha c_{\text{1}}+\alpha c_{\text{2}}+\alpha (\text{2}\alpha -\beta \delta )x_{\text{1}}-\alpha \beta x_{\text{2}}}{\text{4}\alpha -\beta \delta },$$

$$p_{\text{2}}^{NN*}=\frac{\beta \delta (\alpha -\beta \delta )+\beta \delta c_{\text{1}}+\text{2}\alpha c_{\text{2}}-\alpha \beta \delta x_{\text{1}}+\beta (\text{2}\alpha -\beta \delta )x_{\text{2}}}{\text{4}\alpha -\beta \delta },$$

$$D_{\text{1}}^{NN*}=\frac{\text{2}\alpha (\alpha -\beta \delta )-(\text{2}\alpha -\beta \delta )c_{\text{1}}+\alpha c_{\text{2}}+\alpha (\text{2}\alpha -\beta \delta )x_{\text{1}}-\alpha \beta x_{\text{2}}}{\left(\alpha -\beta \delta )(\text{4}\alpha -\beta \delta \right)},$$

$$D_{\text{2}}^{NN*}=\alpha \frac{\beta \delta (\alpha -\beta \delta )+\beta \delta c_{\text{1}}-(\text{2}\alpha -\beta \delta )c_{\text{2}}-\alpha \beta \delta x_{\text{1}}+\beta (\text{2}\alpha -\beta \delta )x_{\text{2}}}{\beta \delta (\alpha -\beta \delta )(\text{4}\alpha -\beta \delta )},$$

$$\pi_{M_{\text{1}}}^{NN*}=\frac{[\text{2}\alpha (\alpha -\beta \delta )-(\text{2}\alpha -\beta \delta )c_{\text{1}}+\alpha c_{\text{2}}+\alpha (\text{2}\alpha -\beta \delta )x_{\text{1}}-\alpha \beta x_{\text{2}}{]}^{\text{2}}}{(\alpha -\beta \delta )(\text{4}\alpha -\beta \delta {)}^{\text{2}}},$$

$$\pi_{M_{\text{2}}}^{NN*}=\alpha \frac{[\beta \delta (\alpha -\beta \delta )+\beta \delta c_{\text{1}}-(\text{2}\alpha -\beta \delta )c_{\text{2}}-\alpha \beta \delta x_{\text{1}}+\beta (\text{2}\alpha -\beta \delta )x_{\text{2}}{]}^{\text{2}}}{\beta \delta (\alpha -\beta \delta )(\text{4}\alpha -\beta \delta {)}^{\text{2}}}.$$

### 4.2 The manufacturer $M_{1}$ adopts live-streaming (Case DN)

In the case where only $M_{\text{1}}$ adopts live streaming, the market is segmented according to consumer type, reflecting how information asymmetry is partially mitigated through signaling [26]. $M_{\text{1}}$’s decision to live-stream serves as a credible signal of its product attributes, reducing uncertainty for a segment of consumers while others remain under imperfect information.

(1)
**The** α(0≤α≤1) **consumers**

Since $M_{\text{1}}$ adopts live-streaming and discloses product information, then those consumers who are well-fit with the product of $M_{\text{1}}$ gain an additional utility *x*_*1*_. Moreover, consumers who purchase from $M_{\text{1}}$ gain a precise valuation $\theta z_{\text{1}}+x_{\text{1}}$ rather than an expected one $\alpha (\theta z_{\text{1}}+x_{\text{1}})$. Those consumers select from the live-streaming channel of $M_{\text{1}}$ and the direct channel of $M_{\text{2}}$, depending on their utilities. In this situation, the utilities obtained by consumers are $U_{\text{1}}=\theta z_{\text{1}}+x_{\text{1}}-p_{\text{1}}$ and $U_{\text{2}}=\beta (\theta z_{\text{2}}+x_{\text{2}})-p_{\text{2}}$, respectively. Thus we have $U_{\text{1}}>U_{\text{2}}>\text{0}$ if $\frac{p_{\text{2}}-\beta x_{\text{2}}}{\beta (p_{\text{1}}-x_{\text{1}})}<\delta <\text{1}$.

(2)
**The** (1−α) **consumers**

Since $M_{\text{1}}$’s product is unfit for those consumers even though $M_{\text{1}}$ discloses the product information, consumers will not buy the product of $M_{\text{1}}$. However, the product information of $M_{\text{2}}$ is unknown. Then those consumers buy $M_{\text{2}}$’s product when they receive a positive utility. The utility for the product of $M_{\text{2}}$ is $U_{\text{2}}=\beta (\theta z_{\text{2}}+x_{\text{2}})-p_{\text{2}}$. In this situation, the market demands for the two products are as follows.


$$D_{\text{1}}=\alpha \frac{\text{1}-\beta \delta +x_{\text{1}}-\beta x_{\text{2}}-p_{\text{1}}+p_{\text{2}}}{\text{1}-\beta \delta }$$ (5)

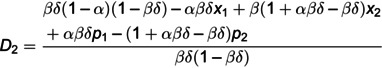

(6)


The two manufacturers engage in Bertrand competition with differentiated products. Their profit functions are:


$$\underset{p_{\text{1}}}{Max}\;\;\pi_{M_{\text{1}}}^{DN}=(p_{\text{1}}-c_{\text{1}})D_{\text{1}}-C_{d\text{1}}=(p_{\text{1}}-c_{\text{1}})\alpha \frac{\text{1}-\beta \delta +x_{\text{1}}-\beta x_{\text{2}}-p_{\text{1}}+p_{\text{2}}}{\text{1}-\beta \delta }-C_{d\text{1}}$$ (7)

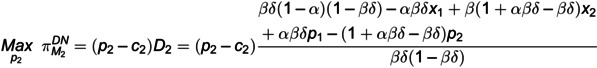

(8)


**Property 2.** *When the manufacturer* $M_{\text{1}}$ *adopts live-streaming,*

(1)
$\pi_{M_{\text{1}}}^{DN}$ *is a concave function with respect to* $p_{\text{1}}$*, indicating there is maximum value for* $\pi_{M_{\text{1}}}^{DN}$.
(2)
$\pi_{M_{\text{2}}}^{DN}$ *is a concave function with respect to* $p_{\text{2}}$*, indicating there is maximum value for* $\pi_{M_{\text{2}}}^{DN}$.

Accordingly, we drive the equilibrium solutions and propose Proposition 2.

**Proposition 2.** *In Case DN, the equilibrium prices, market demands, and profits can be acquired with the conditions* *and* *. The equilibrium solutions are*



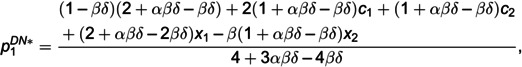




$$p_{\text{2}}^{DN*}=\frac{\beta \delta (\text{2}-\alpha )(\text{1}-\beta \delta )+\alpha \beta \delta c_{\text{1}}+\text{2}(\text{1}+\alpha \beta \delta -\beta \delta )c_{\text{2}}-\alpha \beta \delta x_{\text{1}}+\beta (\text{2}+\alpha \beta \delta -\text{2}\beta \delta )x_{\text{2}}}{\text{4}+\text{3}\alpha \beta \delta -\text{4}\beta \delta },$$

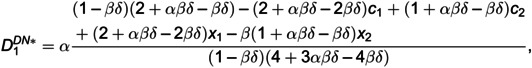





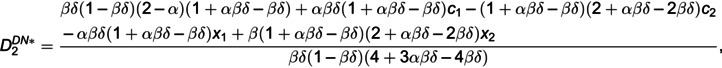





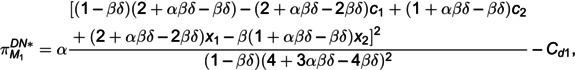




$$\pi_{M_{\text{2}}}^{DN*}=\frac{(\text{1}+\alpha \beta \delta -\beta \delta )[\beta \delta (\text{2}-\alpha )(\text{1}-\beta \delta )+\alpha \beta \delta c_{\text{1}}-(\text{2}+\alpha \beta \delta -\text{2}\beta \delta )c_{\text{2}}-\alpha \beta \delta x_{\text{1}}+\beta (\text{2}+\alpha \beta \delta -\text{2}\beta \delta )x_{\text{2}}{]}^{\text{2}}}{\beta \delta (\text{1}-\beta \delta )(\text{4}+\text{3}\alpha \beta \delta -\text{4}\beta \delta {)}^{\text{2}}}.$$

### 4.3 The manufacturer ${\;M}_{2}$ adopts live-streaming (Case ND)

In the case where only $M_{\text{2}}$ adopts live streaming, the market is segmented based on consumer fit, illustrating how partial information disclosure through live streaming serves as a signaling mechanism [26] and creates an asymmetric information structure across consumer groups.

(1)
**The** β(0≤β≤1) **consumers**

Since $M_{\text{2}}$ adopts live-streaming and discloses product information, then those consumers who are well-fit with $M_{\text{2}}$’s product gain an additional utility *x*_*2*_. Those consumers select from the live-streaming channel of $M_{\text{2}}$ and the direct channel of $M_{\text{1}}$, depending on their utilities $U_{\text{1}}=\alpha (\theta z_{\text{1}}+x_{\text{1}})-p_{\text{1}}$ and $U_{\text{2}}=\theta z_{\text{2}}+x_{\text{2}}-p_{\text{2}}$. Then we have $U_{\text{1}}>U_{\text{2}}>\text{0}\;$ if $\frac{\alpha (p_{\text{2}}-x_{\text{2}})}{p_{\text{1}}-\alpha x_{\text{1}}}<\delta <\text{1}$.

(2)
**The** (1−β) **consumers**

Since $M_{\text{2}}$’s product is unfit for those consumers even though $M_{\text{2}}$ discloses product information, they will not buy the product of $M_{\text{2}}$. However, the product information of $M_{\text{1}}$ is unknown. Then those consumers buy the product of $M_{\text{1}}$ when they receive a positive utility, where the utility for product of $M_{\text{1}}$ is $U_{\text{1}}=\alpha (\theta z_{\text{1}}+x_{\text{1}})-p_{\text{1}}$. In this situation, the market demands for the two products are as follows.



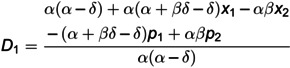

(9)



$$D_{\text{2}}=\beta \frac{-\alpha \delta x_{\text{1}}+\alpha x_{\text{2}}+\delta p_{\text{1}}-\alpha p_{\text{2}}}{\delta (\alpha -\delta )}$$ (10)

The manufacturers engage in Bertrand competition with differentiated products. Their profit-maximization problems are:



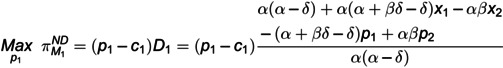

(11)



$$\underset{p_{\text{2}}}{Max}\;\;\pi_{M_{\text{2}}}^{ND}=(p_{\text{2}}-c_{\text{2}})D_{\text{2}}-C_{d\text{2}}=(p_{\text{2}}-c_{\text{2}})\beta \frac{-\alpha \delta x_{\text{1}}+\alpha x_{\text{2}}+\delta p_{\text{1}}-\alpha p_{\text{2}}}{\delta (\alpha -\delta )}-C_{d\text{2}}$$ (12)

**Property 3.** *When the manufacturer*${\;M}_{\text{2}}$ *adopts live-streaming,*

(1) $\pi_{M_{\text{1}}}^{ND}$ *is a concave function with respect to* $p_{\text{1}}$*, indicating there is maximum value for* $\pi_{M_{\text{1}}}^{ND}$.
(2) $\pi_{M_{\text{2}}}^{ND}$ *is a concave function with respect to* $p_{\text{2}}$*, indicating there is maximum value for* $\pi_{M_{\text{2}}}^{ND}$.

Accordingly, we drive the equilibrium solutions and propose Proposition 3.

**Proposition 3.** *In Case DN, the equilibrium prices, market demands, and profits can be acquired with the conditions* α>δ*,* *, and*

*. The equilibrium solutions are*


$$p_{\text{1}}^{ND*}=\frac{\text{2}\alpha (\alpha -\delta )+\text{2}(\alpha +\beta \delta -\delta )c_{\text{1}}+\alpha \beta c_{\text{2}}+\alpha (\text{2}\alpha +\beta \delta -\text{2}\delta )x_{\text{1}}-\alpha \beta x_{\text{2}}}{\left(\text{4}\alpha +\text{3}\beta \delta -\text{4}\delta \right)},$$

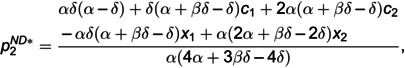




$$D_{\text{1}}^{ND*}=(\alpha +\beta \delta -\delta )\frac{\begin{array}{c} \text{2}\alpha (\alpha -\delta )-(\text{2}\alpha +\beta \delta -\text{2}\delta )c_{\text{1}}+\alpha \beta c_{\text{2}}\\ +\alpha (\text{2}\alpha +\beta \delta -\text{2}\delta )x_{\text{1}}-\alpha \beta x_{\text{2}} \end{array}}{\alpha (\alpha -\delta )(\text{4}\alpha +\text{3}\beta \delta -\text{4}\delta )},$$

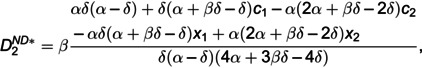





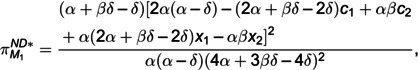





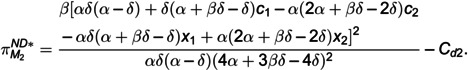



### 4.4 Both manufacturers adopt live-streaming (Case DD)

In the case where both manufacturers adopt live streaming, the market is fully informed due to comprehensive product disclosure by both firms. This represents a scenario where information asymmetry is substantially reduced through dual signaling [26], allowing consumers to make decisions based on actual rather than expected utility. The consumer population is partitioned into four distinct segments based on product fit:

(1)
**The** αβ **consumers**

Since both $M_{\text{1}}$ and $M_{\text{2}}$ adopt live-streaming and disclose product information, then those consumers who are well-fit with the product of $M_{\text{1}}$ and $M_{\text{2}}$ gain an additional utility *x*_*1*_ and *x*_*2*_, respectively. Those consumers select from the $M_{\text{1}}$ and $M_{\text{2}}$ depending on their utilities, where the utilities are $U_{\text{1}}=\theta z_{\text{1}}+x_{\text{1}}-p_{\text{1}}$ and $U_{\text{2}}=\theta z_{\text{2}}+x_{\text{2}}-p_{\text{2}}$. Then we have $U_{\text{1}}>U_{\text{2}}>\text{0}$ if $\frac{p_{\text{2}}-x_{\text{2}}}{p_{\text{1}}-x_{\text{1}}}<\delta <\text{1}$.

(2)
**The** (α−αβ) **consumers**

The (α−αβ) type consumers represent those who are only well-fit with the product of $M_{\text{1}}$ but not simultaneously well-fit with the product of $M_{\text{1}}$ and $M_{\text{2}}$. Then those consumers will not buy the product of $M_{\text{2}}$ since $M_{\text{2}}$ has disclosed product information but does not fit. However, they will buy the product of $M_{\text{1}}$ since $M_{\text{1}}$ has disclosed product information and is well-fit with them. The utility is $U_{\text{1}}=\theta z_{\text{1}}+x_{\text{1}}-p_{\text{1}}$.

(3)
**The** (β−αβ) **consumers**

Similarly, the (β−αβ) type consumers represent those who are only well-fit with the product of $M_{\text{2}}$ but not simultaneously well-fit with the product of $M_{\text{1}}$ and $M_{\text{2}}$. Then those consumers will not buy the product of $M_{\text{1}}$ since $M_{\text{1}}$ has disclosed product information but does not fit. While they will buy the product of $M_{\text{2}}$ since $M_{\text{2}}$ has disclosed product information and is well-fit with them. The utility is $U_{\text{2}}=\theta z_{\text{2}}+x_{\text{2}}-p_{\text{2}}$.

(4)
**The** (1−α−β+αβ) **consumers**

Both the products of $M_{\text{1}}$ and $M_{\text{2}}$ do not fit consumers, as such, they will buy neither $M_{\text{1}}$ nor $M_{\text{2}}$.

In this situation, the market demands for the two products are as follows.



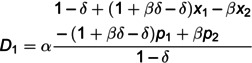

(13)




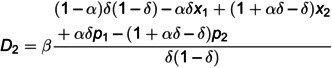

(14)


The manufacturers engage in Bertrand competition. Their profit-maximization problems are:



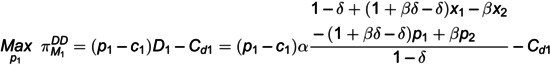

(15)




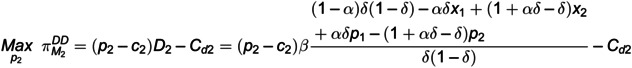

(16)


**Property 4.** *When both manufacturers adopt live-streaming,*

(1) $\pi_{M_{\text{1}}}^{DD}$ *is a concave function with respect to* $p_{\text{1}}$*, indicating there is maximum value for* $\pi_{M_{\text{1}}}^{DD}$.
(2) $\pi_{M_{\text{2}}}^{DD}$ *is a concave function with respect to* $p_{\text{2}}$*, indicating there is maximum value for* $\pi_{M_{\text{2}}}^{DD}$.

Accordingly, we drive the equilibrium solutions and propose Proposition 4.

**Proposition 4.** *In Case DD, the equilibrium prices, market demands, and profits can be acquired with the conditions* *,*

*. The equilibrium solutions are*



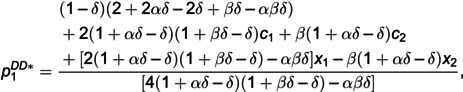





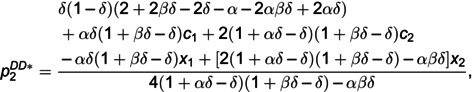




$$D_{\text{1}}^{DD*}=\alpha (\text{1}+\beta \delta -\delta )\frac{\begin{array}{c} \left(\text{1}-\delta )(\text{2}+\text{2}\alpha \delta -\text{2}\delta +\beta \delta -\alpha \beta \delta \right)\\ -\left[\text{2}(\text{1}+\alpha \delta -\delta )(\text{1}+\beta \delta -\delta )-\alpha \beta \delta \right]c_{\text{1}}+\beta (\text{1}+\alpha \delta -\delta )c_{\text{2}}\\ +\left[\text{2}(\text{1}+\alpha \delta -\delta )(\text{1}+\beta \delta -\delta )-\alpha \beta \delta \right]x_{\text{1}}-\beta (\text{1}+\alpha \delta -\delta )x_{\text{2}} \end{array}}{(\text{1}-\delta )\left[\text{4}(\text{1}+\alpha \delta -\delta )(\text{1}+\beta \delta -\delta )-\alpha \beta \delta \right]},$$

$$D_{\text{2}}^{DD*}=\beta (\text{1}+\alpha \delta -\delta )\frac{\begin{array}{c} \delta (\text{1}-\delta )(\text{2}+\text{2}\beta \delta -\text{2}\delta -\alpha -\text{2}\alpha \beta \delta +\text{2}\alpha \delta )\\ +\alpha \delta (\text{1}+\beta \delta -\delta )c_{\text{1}}-\left[\text{2}(\text{1}+\alpha \delta -\delta )(\text{1}+\beta \delta -\delta )-\alpha \beta \delta \right]c_{\text{2}}\\ -\alpha \delta (\text{1}+\beta \delta -\delta )x_{\text{1}}+\left[\text{2}(\text{1}+\alpha \delta -\delta )(\text{1}+\beta \delta -\delta )-\alpha \beta \delta \right]x_{\text{2}} \end{array}}{\delta (\text{1}-\delta )\left[\text{4}(\text{1}+\alpha \delta -\delta )(\text{1}+\beta \delta -\delta )-\alpha \beta \delta \right]},$$

$$\pi_{M_{\text{1}}}^{DD*}=\alpha (\text{1}+\beta \delta -\delta )\frac{\begin{array}{c} \left\{(\text{1}-\delta )(\text{2}+\text{2}\alpha \delta -\text{2}\delta +\beta \delta -\alpha \beta \delta \right)\\ -\left[\text{2}(\text{1}+\alpha \delta -\delta )(\text{1}+\beta \delta -\delta )-\alpha \beta \delta \right]c_{\text{1}}+\beta (\text{1}+\alpha \delta -\delta )c_{\text{2}}\\ +\left[\text{2}(\text{1}+\alpha \delta -\delta )(\text{1}+\beta \delta -\delta )-\alpha \beta \delta \right]x_{\text{1}}-\beta (\text{1}+\alpha \delta -\delta )x_{\text{2}}{\}}^{\text{2}} \end{array}}{(\text{1}-\delta )[\text{4}(\text{1}+\alpha \delta -\delta )(\text{1}+\beta \delta -\delta )-\alpha \beta \delta {]}^{\text{2}}}-C_{d\text{1}},$$

$$\pi_{M_{\text{2}}}^{DD*}=\beta (\text{1}+\alpha \delta -\delta )\frac{\begin{array}{c} \left\{\delta (\text{1}-\delta )(\text{2}+\text{2}\beta \delta -\text{2}\delta -\alpha -\text{2}\alpha \beta \delta +\text{2}\alpha \delta \right)\\ +\alpha \delta (\text{1}+\beta \delta -\delta )c_{\text{1}}-\left[\text{2}(\text{1}+\alpha \delta -\delta )(\text{1}+\beta \delta -\delta )-\alpha \beta \delta \right]c_{\text{2}}\\ -\alpha \delta (\text{1}+\beta \delta -\delta )x_{\text{1}}+\left[\text{2}(\text{1}+\alpha \delta -\delta )(\text{1}+\beta \delta -\delta )-\alpha \beta \delta \right]x_{\text{2}}{\}}^{\text{2}} \end{array}}{\delta (\text{1}-\delta )[\text{4}(\text{1}+\alpha \delta -\delta )(\text{1}+\beta \delta -\delta )-\alpha \beta \delta {]}^{\text{2}}}-C_{d\text{2}}.$$

## 5. Model analysis

### 5.1 The operational strategy of competitive manufacturers

**Property 5**. *The impact of the additional utility x*_*1*_ *provided by* $M_{\text{1}}$ *on equilibrium outcomes under all four scenarios (j = NN, DN, ND, DD) is as follows:*

(1) *Pricing strategy:* $\frac{\partial p_{\text{1}}^{j*}}{\partial x_{\text{1}}}>\text{0}$, $\frac{\partial p_{\text{2}}^{j*}}{\partial x_{\text{1}}}<\text{0}$.
(2) *Market demand:* $\frac{\partial D_{\text{1}}^{j*}}{\partial x_{\text{1}}}>\text{0}$, $\frac{\partial D_{\text{2}}^{j*}}{\partial x_{\text{1}}}<\text{0}$.
(3) *Profit:* $\frac{\partial \pi_{M_{\text{1}}}^{j*}}{\partial x_{\text{1}}}>\text{0}$*,* $\frac{\partial \pi_{M_{\text{2}}}^{j*}}{\partial x_{\text{1}}}<$0*.*

Property 5 indicates that when consumers derive higher additional utility (*x*_*1*_) from using $M_{\text{1}}^{\prime }s$ product (due to better fit, superior experience, or enhanced trust), $M_{\text{1}}\;$can command a higher price while still attracting more demand. This happens because increased perceived value enhances consumers’ willingness to pay. In response, $M_{\text{2}}$ is forced to lower its price to remain competitive, yet still loses market share and profit.

**Property 6**. *The impact of the additional utility x*_*2*_ *provided by* $M_{\text{2}}$ *on equilibrium outcomes under all four scenarios (j = NN, DN, ND, DD) is as follows:*

(1) *Pricing strategy:* $\frac{\partial p_{\text{1}}^{j*}}{\partial x_{\text{2}}}<\text{0}$, $\frac{\partial p_{\text{2}}^{j*}}{\partial x_{\text{2}}}>\text{0}$.
(2) *Market demand:* $\frac{\partial D_{\text{1}}^{j*}}{\partial x_{\text{2}}}<\text{0}$, $\frac{\partial D_{\text{2}}^{j*}}{\partial x_{\text{2}}}>\text{0}$.
(3) *Profit:* $\frac{\partial \pi_{M_{\text{1}}}^{j*}}{\partial x_{\text{2}}}<\text{0}$, $\frac{\partial \pi_{M_{\text{2}}}^{j*}}{\partial x_{\text{2}}}>\text{0}$.

Property 6 reveals a symmetric effect to that observed in Property 5: when consumers gain higher additional utility (*x*_*2*_) from $M_{\text{2}}^{\prime }s$ product, $M_{\text{2}}$ can raise its price while still expanding market demand and profit. This advantage forces $M_{\text{1}}$ to lower its price in response, yet $M_{\text{1}}$ still ends up with reduced market share and profitability.

Furthermore, from Properties 5 and 6, for competing manufacturers, enhancing the perceived additional utility that consumers receive from a product (such as through better product-match communication, service quality, or experiential value) directly strengthens pricing power, market share, and profit margins. In a competitive context, when one manufacturer significantly improves consumer utility, rivals cannot rely solely on price reductions to remain competitive. Instead, they should focus on elevating their own product value or live-streaming presentation quality. Besides, increasing the fit-based utility (*x*_*1*_ or *x*_*2*_) is a powerful competitive lever. Manufacturers should prioritize strategies that enhance perceived suitability and consumer trust (such as personalized demonstrations, trial use campaigns, or stronger after-sales support) to build sustainable advantage in live-streaming markets. These findings underscore that in live-streaming commerce, non-price advantages rooted in product suitability and consumer experience play a decisive role in shaping competitive outcomes.

**Property 7.** *The impact of the consumer fit proportion* α *on equilibrium pricing strategies is as follows:*

(1) $M_{\text{1}}\;{\;}^{\prime }s\;$ *pricing:* $\frac{\partial p_{\text{1}}^{NN*}}{\partial \alpha }>\text{0}$, $\frac{\partial p_{\text{1}}^{DN*}}{\partial \alpha }<\text{0}$, $\frac{\partial p_{\text{1}}^{ND*}}{\partial \alpha }>\text{0}$, $\frac{\partial p_{\text{1}}^{DD*}}{\partial \alpha }<\text{0}$.
(2) $M_{\text{2}}\;{\;}^{\prime }s\;$ *pricing:* $\frac{\partial p_{\text{2}}^{NN*}}{\partial \alpha }<\text{0}$, $\frac{\partial p_{\text{2}}^{DN*}}{\partial \alpha }<\text{0}$, $\frac{\partial p_{\text{2}}^{ND*}}{\partial \alpha }<\text{0}$, $\frac{\partial p_{\text{2}}^{DD*}}{\partial \alpha }<\text{0}$.

Property 7(1) reveals that the effect of α (the proportion of consumers suited to $M_{\text{1}}{}^{\prime }\;s$ product) on $M_{\text{1}}{}^{\prime }\;s$ optimal price depends critically on whether $M_{\text{1}}$ uses live streaming. In Case NN and ND ($M_{\text{1}}$ does not live-stream), a higher α allows $M_{\text{1}}$ to raise its price. Without detailed product information, consumers who naturally prefer $M_{\text{1}}$ are more likely to purchase directly, giving $M_{\text{1}}$ market power. In Case DN and DD ($M_{\text{1}}$ does live-stream), a higher α leads $M_{\text{1}}$ to lower its price. Despite high innate preference, full information disclosure may reveal misfits, prompting $M_{\text{1}}$ to use price cuts to retain potentially hesitant customers. By contrast, Property 7(2) shows that $M_{\text{2}}$ always lowers its price as α increases. A stronger innate preference for $M_{\text{1}}$ forces $M_{\text{2}}$ to compete more aggressively on price to attract any remaining customers.

**Property 8.** *The impact of consumer fit proportion* β *(suitability for M₂‘s product) on equilibrium pricing is as follows:*

(1) $M_{\text{1}}{}^{\prime }\;s\;$ *pricing:* $\frac{\partial p_{\text{1}}^{NN*}}{\partial \beta }<\text{0}$, $\frac{\partial p_{\text{1}}^{DN*}}{\partial \beta }<\text{0}$, $\frac{\partial p_{\text{1}}^{ND*}}{\partial \beta }<\text{0}$, $\frac{\partial p_{\text{1}}^{DD*}}{\partial \beta }<\text{0}$.
(2) $M_{\text{2}}{}^{\prime }\;s\;$ *pricing:* $\frac{\partial p_{\text{2}}^{NN*}}{\partial \beta }>\text{0}$, $\frac{\partial p_{\text{2}}^{DN*}}{\partial \beta }>\text{0}$, $\frac{\partial p_{\text{2}}^{ND*}}{\partial \beta }<\text{0}$, $\frac{\partial p_{\text{2}}^{DD*}}{\partial \beta }<\text{0}$.

Property 8 reveals how the proportion of consumers suited to $M_{\text{2}}{}^{\prime }\;s$ product (β) affects both firms’ pricing strategies. $M_{\text{1}}$ consistently lowers its price as β increases across all four scenarios. This reflects $M_{\text{1}}{}^{\prime }\;s$ need to compete more aggressively when more consumers are inherently suited to $M_{\text{2}}{}^{\prime }\;s$ product. $M_{\text{2}}{}^{\prime }\;s$ response depends on its information disclosure strategy. When $M_{\text{2}}$ does not live-stream (Cases NN and DN), it can raise prices as β increases, exploiting its natural advantage without full information disclosure. When $M_{\text{2}}$ does live-stream (Cases ND and DD), it lowers prices despite high β, because detailed product information may reveal misfits even to naturally suited consumers.

Properties 7 and 8 together offer key insights into pricing and disclosure strategies in competitive markets. When consumer fit is high but product information is limited, manufacturers gain pricing power since information asymmetry enables them to capitalize on innate preference without reducing prices. In contrast, facing a competitor with strong consumer fit may necessitate price cuts, particularly for manufacturers with weaker inherent appeal. Live streaming, however, changes this dynamic: even with high fit, full information disclosure can weaken a manufacturer’s ability to maintain high prices, as greater transparency may make consumers more cautious unless prices are lowered. Therefore, optimal pricing depends not only on consumer preferences but also on the level of product information revealed. Manufacturers should anticipate pricing adjustments in response to rivals’ fit advantages and disclosure strategies. Emphasizing unique product attributes—rather than competing on price alone—can help maintain profit margins and enhance market positioning.

**Fig 1:**
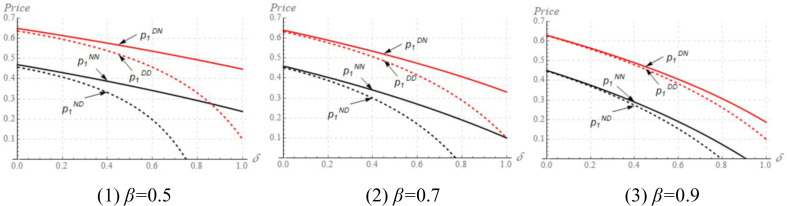
The impact of *δ* on the M_1_’s pricing strategy in different cases.

**Fig 2:**
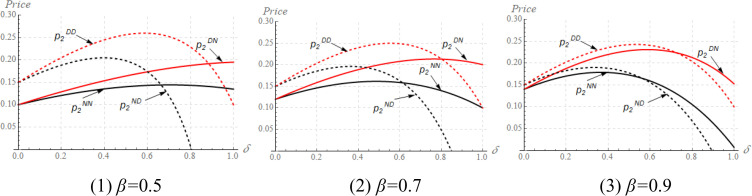
The impact of *δ* on the M_2_’s pricing strategy in different cases.

The impact of *δ* on equilibrium solutions cannot be proved analytically, we thus turn to numerical studies. We fix *x*_*1*_ = *x*_*2*_ = 0.2, *c*_*1*_ = *c*_*2*_ = 0.1, *α* = 0.7, and vary *δ*, *α*, *β* to study the mechanism, then we plot Figs 1 and 2.

Fig 1 shows that the higher the *δ*, the lower the price set by M_1_. A high *δ* indicates the quality of the M_2_’s product becomes higher. In this situation, M_1_ should decrease its price to compete with M_2_. However, Fig 2 shows that the changing rule of the M_2_’s price is quite different from that of the M_1_. More precisely, when *δ* is low, the M_2_’s price increases with *δ*; while the M_2_’s price decreases with *δ* when *δ* belongs to a high value. In a low *δ*, the M_1_’s price is at a high level and thus M_2_ could design a high price under a channel competitive environment. Nevertheless, when *δ* belongs to a high value, M_1_ designs a low price even though its product has a quality advantage, namely, z_1_(=1)>z_2_(=*δ*). In this context, M_2_ should also reduce its price to maintain the competitiveness of its product, leading to the result that M_2_’s price decreases with *δ* when *δ* is in a high value. Obviously, a high quality of M_2_’s product does not necessarily allow M_2_ to raise the price. Rather, M_2_ should refer to the pricing strategy of the manufacturer who has a quality advantage (i.e., M_1_) in determining the price.

### 5.2 The opening strategy of live-streaming channel

In this section, we compare the equilibrium price and profit of M_1_ and M_2_ to study the impact of adopting a live-streaming channel.

**Proposition 5.** *The equilibrium prices under the four scenarios satisfy the following relationships:*

(1) *The price differential for M*_*1*_: $p_{\text{1}}^{ND*}<p_{\text{1}}^{NN*}<p_{\text{1}}^{DD*}<p_{\text{1}}^{DN*}$.
(2) *The price differential for M*_*2*_: $p_{\text{2}}^{DN*}<p_{\text{2}}^{NN*}<p_{\text{2}}^{DD*}<p_{\text{2}}^{ND*}$.

Proposition 5 indicates that, first, when a manufacturer opens a live streaming channel, it can charge higher prices, namely, $p_{\text{1}}^{DD*}>p_{\text{1}}^{ND*}$ and $p_{\text{1}}^{DN*}>p_{\text{1}}^{NN*}$ for M_1_; $p_{\text{2}}^{DD*}>p_{\text{2}}^{DN*}$ and $p_{\text{2}}^{ND*}>p_{\text{2}}^{NN*}$ for M_2_. This occurs because live streaming provides authentic product information, allowing consumers to better understand product value. With reduced uncertainty, manufacturers can command price premiums. Second, a case of unilateral opening live-streaming channel leads to the highest price for the manufacturer, namely, ${p_{\text{1}}^{DN*}>max\{p}_{\text{1}}^{ND*},p_{\text{1}}^{NN*},p_{\text{1}}^{DD*}\}$ and ${p_{\text{2}}^{ND*}>max\{p}_{\text{2}}^{DN*},p_{\text{2}}^{NN*},p_{\text{2}}^{DD*}\}$. This represents a “first-mover advantage” in information disclosure—the streaming manufacturer gains significant pricing power while competitors lack equivalent means to demonstrate value. Thus, a manufacturer facing weak competition should consider unilateral live streaming to maximize margins before competitors adopt similar strategies. Third, when both manufacturers stream, prices decrease compared to unilateral streaming, namely, $p_{\text{1}}^{DD*}<p_{\text{1}}^{DN*}$ and $p_{\text{1}}^{ND*}<p_{\text{1}}^{NN*}$ for M_1_; $p_{\text{2}}^{DD*}<p_{\text{2}}^{ND*}$ and $p_{\text{2}}^{DN*}<p_{\text{2}}^{NN*}$ for M_2_. This reflects intensified competition—when both sides provide full information, consumers can make direct comparisons, forcing manufacturers to compete more aggressively on price.

Proposition 5 further indicates that in mature markets where live streaming is widely used by competitors, manufacturers should focus on product differentiation rather than relying solely on information disclosure to maintain prices. To use live streaming effectively, firms should adopt it early to gain a pricing advantage, anticipate price erosion as rivals follow suit, combine live streaming with distinctive product features to sustain premiums, and monitor competitors’ live streaming activities given their direct impact on pricing flexibility. Overall, the value of live streaming depends critically on competitive dynamics—it offers the greatest advantage when implemented before competitors.

**Proposition 6.** *The comparison of* $M_{\text{1}}$*’s equilibrium profits across scenarios reveals:*

(1) *Case NN vs Case DN* : 

$\pi_{M_{\text{1}}}^{NN*}<\pi_{M_{\text{1}}}^{DN*}$ *if* $C_{d\text{1}}<\tilde{C_{d\text{1}}}$*, where* .

(2) *Case ND vs Case DD* : 

$\pi_{M_{\text{1}}}^{ND*}<\pi_{M_{\text{1}}}^{DD*}$ *if* $C_{d\text{1}}<\hat{C_{d\text{1}}}$*, where*


$$\begin{array}{c} \hat{C_{d\text{1}}}=\alpha (\text{1}+\beta \delta -\delta )\frac{\begin{array}{c} \left\{(\text{1}-\delta )(\text{2}+\text{2}\alpha \delta -\text{2}\delta +\beta \delta -\alpha \beta \delta \right)\\ -\left[\text{2}(\text{1}+\alpha \delta -\delta )(\text{1}+\beta \delta -\delta )-\alpha \beta \delta \right]c_{\text{1}}+\beta (\text{1}+\alpha \delta -\delta )c_{\text{2}}\\ +\left[\text{2}(\text{1}+\alpha \delta -\delta )(\text{1}+\beta \delta -\delta )-\alpha \beta \delta \right]x_{\text{1}}-\beta (\text{1}+\alpha \delta -\delta )x_{\text{2}}{\}}^{\text{2}} \end{array}}{(\text{1}-\delta )[\text{4}(\text{1}+\alpha \delta -\delta )(\text{1}+\beta \delta -\delta )-\alpha \beta \delta {]}^{\text{2}}}\\ -\frac{\begin{array}{c} (\alpha +\beta \delta -\delta )[\text{2}\alpha (\alpha -\delta )-(\text{2}\alpha +\beta \delta -\text{2}\delta )c_{\text{1}}+\alpha \beta c_{\text{2}}\\ +\alpha (\text{2}\alpha +\beta \delta -\text{2}\delta )x_{\text{1}}-\alpha \beta x_{\text{2}}{]}^{\text{2}} \end{array}}{\alpha (\alpha -\delta )(\text{4}\alpha +\text{3}\beta \delta -\text{4}\delta {)}^{\text{2}}}. \end{array}$$

Proposition 6 shows that the opening of the live-streaming channel does not necessarily increase the profit of $M_{\text{1}}$, and it happens only if the operational cost for the live-streaming channel is relatively low. Otherwise, $M_{\text{1}}$ may be worse off if the operational cost for the live-streaming channel is relatively high. Generally, when the operational cost is high enough to cover the benefit generated from the live-streaming channel, the opening of the live-streaming channel becomes uneconomic.

**Proposition 7.** *The comparison of* $M_{\text{2}}$*’s equilibrium profits across scenarios reveals:*

(1) *Case NN vs Case ND* : 

$\pi_{M_{\text{2}}}^{NN*}<\pi_{M_{\text{2}}}^{ND*}$ *if* $C_{d\text{2}}<\tilde{C_{d\text{2}}}$*, where*



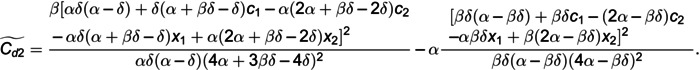

(2) *Case DN vs Case DD* : 

$\pi_{M_{\text{2}}}^{DN*}<\pi_{M_{\text{2}}}^{DD*}$ *if* $C_{d\text{2}}<\hat{C_{d\text{2}}}$*, where*


$$\begin{array}{c} \hat{C_{d\text{2}}}=\beta (\text{1}+\alpha \delta -\delta )\frac{\begin{array}{c} \left\{\delta (\text{1}-\delta )(\text{2}+\text{2}\beta \delta -\text{2}\delta -\alpha -\text{2}\alpha \beta \delta +\text{2}\alpha \delta \right)\\ +\alpha \delta (\text{1}+\beta \delta -\delta )c_{\text{1}}-\left[\text{2}(\text{1}+\alpha \delta -\delta )(\text{1}+\beta \delta -\delta )-\alpha \beta \delta \right]c_{\text{2}}\\ -\alpha \delta (\text{1}+\beta \delta -\delta )x_{\text{1}}+\left[\text{2}(\text{1}+\alpha \delta -\delta )(\text{1}+\beta \delta -\delta )-\alpha \beta \delta \right]x_{\text{2}}{\}}^{\text{2}} \end{array}}{\delta (\text{1}-\delta )[\text{4}(\text{1}+\alpha \delta -\delta )(\text{1}+\beta \delta -\delta )-\alpha \beta \delta {]}^{\text{2}}}\\ -\frac{\begin{array}{c} (\text{1}+\alpha \beta \delta -\beta \delta )[\beta \delta (\text{2}-\alpha )(\text{1}-\beta \delta )+\alpha \beta \delta c_{\text{1}}\\ -(\text{2}+\alpha \beta \delta -\text{2}\beta \delta )c_{\text{2}}-\alpha \beta \delta x_{\text{1}}+\beta (\text{2}+\alpha \beta \delta -\text{2}\beta \delta )x_{\text{2}}{]}^{\text{2}} \end{array}}{\beta \delta (\text{1}-\beta \delta )(\text{4}+\text{3}\alpha \beta \delta -\text{4}\beta \delta {)}^{\text{2}}}. \end{array}$$

Proposition 7 confirms that the profitability of live streaming depends critically on cost management, $M_{\text{2}}$ only benefits from live-streaming adoption when operational costs remain below specific threshold levels. This mirrors the finding for $M_{\text{1}}$ in Proposition 6, highlighting a universal principle in live-streaming strategy.

Therefore, prior to committing to live-streaming investments, manufacturers should perform a systematic cost-benefit analysis to verify whether anticipated revenues justify operational expenditures and generate positive net value. A successful live-streaming strategy depends not only on content quality but on establishing a sustainable cost-benefit advantage. Through strategic cost management, manufacturers can achieve enduring competitive differentiation; conversely, excessive investment can erode profit margins despite driving sales growth.

**Fig 3:**
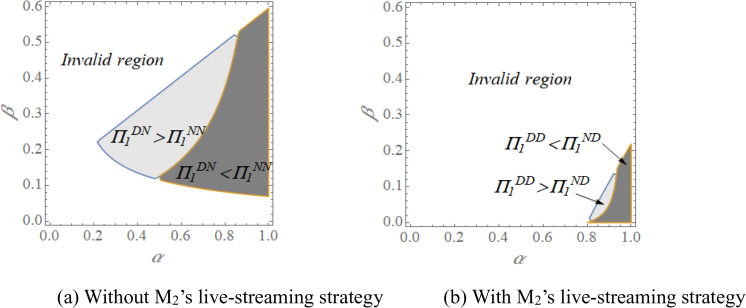
The opening strategy of live-streaming channel for$M_{1}$ with varying *α* and *β.*

**Fig 4:**
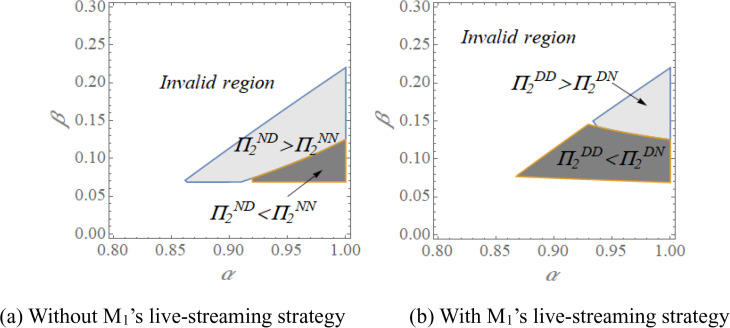
The opening strategy of live-streaming channel for$M_{2}$ with varying *α* and *β.*

Note that there are some factors relevant to consumer features that influence the live-streaming strategy. Given the complexity of deriving closed-form equilibrium solutions, we employ numerical simulation to analyze manufacturer profitability. Using the baseline values *x*_*1*_ = *x*_*2*_ = 0.5, *C*_*d1*_ = *C*_*d2*_ = 0.05, *c*_*1*_ = 0.1, *c*_*2*_ = 0.05, *δ* = 0.8, we systematically vary the consumer fit parameters *α* and *β*. The resulting profit patterns are presented in Figs 3 and 4.

Fig 3 reveals that the profitability of $M_{\text{1}}$’s live-streaming strategy depends critically on consumer fit parameters *α* and *β*, a finding that can be effectively interpreted through established psychological frameworks. In the absence of $M_{\text{2}}$’s live-streaming strategy (i.e., Fig 3(a)), $M_{\text{1}}$ benefits from the live-streaming when *α* and *β* belong to a median value. From the perspective of social presence theory, this enables live streaming to establish meaningful social presence while avoiding excessive cognitive load on consumers. As a result, it can retain consumers who access detailed product information through live-streaming while still capturing those with inherent preference for $M_{\text{1}}$’s products. The observed negative impact at high *α* levels aligns with principles of parasocial interaction theory. When consumers already maintain strong product affinity (high *α*), detailed exposure through live streaming may disrupt rather than strengthen existing psychological bonds, as they gain awareness of potential mismatches that were previously overlooked. This phenomenon reflects an “uncanny valley” effect in consumer engagement, where excessive transparency can undermine carefully constructed brand perceptions, potentially causing decision paralysis among previously loyal customers. Therefore, a high enough *α* may hurt $M_{\text{1}}$.

When $M_{\text{2}}$ employs live streaming (i.e., Fig 3(b)), $M_{\text{1}}$ benefits from live-streaming only when *α* is moderate and *β* is sufficiently low. This pattern aligns with social comparison theory: in dual-streaming environments, consumers naturally compare offerings, making $M_{\text{1}}$’s live-streaming most effective against clearly inferior alternatives (low *β*). Conversely, $M_{\text{1}}$ suffers losses from live-streaming when *α* is high and *β* is low. Unlike scenarios without $M_{\text{2}}$’s live-streaming, the intensified channel competition demands even lower *β* values for $M_{\text{1}}$ to profit. This occurs because only when $M_{\text{2}}$’s product demonstrates substantially weaker consumer appeal can $M_{\text{1}}$ capture sufficient market share through live-streaming to offset competitive pressures. This analysis reveals that under competitive live-streaming conditions, success depends not only on a manufacturer’s own product appeal but crucially on the relative disadvantage of competing products.

Concerning the strategy of $M_{\text{2}}$, Fig 4 shows that in the absence of $M_{\text{1}}$’s live-streaming strategy (i.e., Fig 4(a)), $M_{\text{2}}$ derives a higher profit from live-streaming when *α* is large and *β* belongs to a median value. This pattern aligns with social presence theory, in the absence of competing live-streaming, $M_{\text{2}}$’s live-streaming creates a distinctive social environment that attracts consumers who are naturally inclined toward $M_{\text{1}}$’s products but remain open to alternatives. The live-streaming experience provides the human warmth and immediacy that helps overcome initial preference barriers. While $M_{\text{2}}$ will be worse off by opening the live-streaming if *α* is large enough and *β* is low. This scenario reflects principles of customer engagement theory, when $M_{\text{2}}$’s product has low inherent appeal (low *β*), live-streaming exposure may actually reinforce negative perceptions rather than building engagement. Consumers comparing $M_{\text{2}}$’s clearly inferior product against $M_{\text{1}}$’s well-suited offering through live-streaming demonstrations experience cognitive dissonance that further diminishes purchase intention.

In the presence of $M_{\text{1}}$’s live-streaming strategy (i.e., Fig 4(b)), $M_{\text{2}}$ benefits from live-streaming when *α* and *β* belong to a high value, while $M_{\text{2}}$ is hurt by the live-streaming when *α* is high and *β* is low. From the perspective of social comparison theory, the dual-streaming context creates a natural comparison environment where consumers simultaneously evaluate both products. A high *β* enables $M_{\text{2}}$ to withstand this direct comparison, as its product demonstrates sufficient inherent appeal to compete effectively against $M_{\text{1}}$’s offering. The scenario where $M_{\text{2}}$ suffers despite high *α* and low *β* can be understood through decision avoidance theory. When consumers recognize $M_{\text{2}}$’s product as clearly inferior (low *β*) compared to $M_{\text{1}}$’s well-suited alternative (high *α*), the additional information provided through live-streaming may actually trigger decision avoidance rather than facilitating choice. The live-streaming exposure makes the quality disparity more apparent, leading consumers to defer purchase decisions altogether.

Totally, the opening of the live-streaming channel does not definitely increase the profit of manufacturers. Rather, a situation with a median preference for the manufacturer’s product is suitable to introduce the live-streaming. In this sense, if the competitor adopts live-streaming strategy, a weaker product preference for the competitor’s product is needed to allow the current manufacturer who has a quality advantage to derive higher profit; while a stronger product preference for the competitor’s product is needed to satisfy to allow the current manufacturer who has quality disadvantage to derive higher profit.

## 6. Conclusion

This study employs game-theoretic modeling and numerical analysis to systematically examine the live-streaming strategies of two competing manufacturers offering quality-differentiated products. The findings indicate that live streaming is not universally effective as a marketing tool; rather, its success depends significantly on contextual factors including product positioning, competitive dynamics, and consumer characteristics.

The study reveals that the success of live-streaming strategies fundamentally depends on the product’s inherent value proposition. Live streaming primarily amplifies existing value perceptions through enhanced information transparency, rather than creating new value foundations. Consequently, only products with favorable price-value ratios can gain competitive advantage through live-streaming; products with poor price-value ratios tend to expose their deficiencies in live-streaming environments, ultimately weakening their market position. Specifically, while live-streaming enables manufacturers to secure pricing advantages, simultaneous competitor adoption intensifies channel competition and exerts downward pressure on prices. Regarding profitability, high-quality manufacturers achieve optimal results when facing no competitor live-streaming and when both their own and competitors’ consumer compatibility parameters fall within moderate ranges. Under competitive live-streaming conditions, they require moderate compatibility combined with low competitor compatibility to maintain profitability. For low-quality manufacturers, maximum benefits occur with high competitor compatibility and moderate self- compatibility without competitor live-streaming, whereas competitive live-streaming environments necessitate high values for both parameters to achieve profitability.

At the operational level, this study reveals complex pricing dynamics. While high product compatibility typically supports premium pricing strategies, we find that live-streaming significantly alters traditional pricing patterns. When consumer compatibility generates substantial additional utility, manufacturers can raise prices to increase profits, forcing competitors to reduce prices and consequently lose market share and profitability. However, the introduction of live streaming modifies this dynamic. Prior to live-streaming adoption, higher product compatibility strengthens manufacturers’ pricing power. After implementing live-streaming, however, even highly compatible products may require price reductions to maintain consumer engagement. Notably, competitors’ prices consistently decline as a manufacturer’s product compatibility increases. Furthermore, quality improvements by lower-quality manufacturers compel high-quality competitors to lower their prices, while lower-quality manufacturers themselves exhibit an inverted U-shaped pricing relationship with quality improvement—prices initially rise but eventually decline with further quality enhancements.

Theoretically, this study frames live streaming as a signaling mechanism that conveys product quality and fit information [26], showing how it mitigates information asymmetry by transforming experience attributes into searchable characteristics [38], while also extending Bertrand competition frameworks to incorporate information disclosure strategies in differentiated markets. By further integrating social presence theory and customer engagement frameworks, we establish how live-streaming creates value through psychological mechanisms that extend beyond mere information transmission.

These findings offer actionable guidance for manufacturers developing live-streaming strategies. Firms should approach live streaming as a strategic investment rather than a compliance-driven marketing activity. High-quality manufacturers are advised to adopt live streaming early in less competitive scenarios, targeting moderately compatible consumer segments with cost-effective formats. Lower-quality manufacturers should focus on inherently attractive niche markets, establish clear value propositions before investing in live streaming, and leverage it to demonstrate comparative advantages. Critically, all manufacturers must conduct rigorous cost-benefit analyses, as live streaming enhances profitability only when operational costs remain below context-specific thresholds.

While this study offers valuable insights, several limitations merit attention and suggest productive directions for future research. First, our model assumes fully rational, utility-maximizing consumers and examines static competition in a duopoly market. This framework does not incorporate important psychological factors such as emotional influences and brand loyalty, nor does it account for dynamic market elements. The model could be extended to examine platform-manufacturer coopetition in multi-brand environments, product return policies specific to live streaming commerce, and cross-product promotional spillovers within brand portfolios. Substantial opportunities also exist for investigating how live streaming content quality, platform recommendation algorithms, and dynamic brand-building processes affect strategic outcomes. Future work could likewise analyze variable cost structures, such as commission-based pricing—and conduct empirical validation using real-world data from major live streaming platforms. Finally, integrating behavioral perspectives, including how consumer trust and emotional engagement develop through live streaming interactions—would help bridge operational decision-making with consumer psychology and platform economics, ultimately enriching our understanding of live streaming commerce dynamics.

## Supporting information

S1 File Supplementary data for this article can be found in the Supporting Information files. (PDF)
