# Supplementary material for: The live-streaming strategy for competitive manufacturers considering information disclosure and product heterogeneity
Source: PLoS One. 2026 Feb 11;21(2):e0339997. doi: 10.1371/journal.pone.0339997 (PMC12893614; doi:10.1371/journal.pone.0339997)
Supplement: S1 File — (PDF) [file pone.0339997.s001.pdf]

Appendix for "The live-streaming strategy for competitive manufacturers considering information disclosure and product heterogeneity"

Proof of Property 1.

The first derivative of $\pi_{M_1}^{NN}$ with respect to (w.r.t.) p_1 is

$$\frac{\partial \pi_{M_1}^{NN}}{\partial p_1}=\frac{\alpha-\beta\delta+c_1+\alpha x_1-\beta x_2-2p_1+p_2}{\alpha-\beta\delta}. \tag{A1}$$

The second derivative is $\frac{\partial^2 \pi_{M_1}^{NN}}{\partial p_1^2}=\frac{-2}{\alpha-\beta\delta}<0$. Then $\pi_{M_1}^{NN}$ is a concave function w.r.t. p_1.

The first derivative of $\pi_{M_2}^{NN}$ w.r.t. p_2 is

$$\frac{\partial \pi_{M_2}^{NN}}{\partial p_2}=\frac{\alpha c_2-\alpha\beta\delta x_1+\alpha\beta x_2+\beta\delta p_1-2\alpha p_2}{\beta\delta(\alpha-\beta\delta)}. \tag{A2}$$

The second derivative is $\frac{\partial^2 \pi_{M_2}^{NN}}{\partial p_2^2}=\frac{-2\alpha}{\beta\delta(\alpha-\beta\delta)}$. Then $\pi_{M_2}^{NN}$ is a concave function w.r.t. p_2.

Proof of Proposition 1.

Let Eq.(A1)=0 and Eq.(A2)=0, then we have

$$p_1^{NN*}=\frac{2\alpha(\alpha-\beta\delta)+2\alpha c_1+\alpha c_2+\alpha(2\alpha-\beta\delta)x_1-\alpha\beta x_2}{4\alpha-\beta\delta},$$

$$p_2^{NN*}=\frac{\beta\delta(\alpha-\beta\delta)+\beta\delta c_1+2\alpha c_2-\alpha\beta\delta x_1+\beta(2\alpha-\beta\delta)x_2}{4\alpha-\beta\delta}.$$

Substituting p_1^{NN*} and p_2^{NN*} into Eq.(1) and Eq.(2), then we have

$$D_1^{NN*}=\frac{2\alpha(\alpha-\beta\delta)-(2\alpha-\beta\delta)c_1+\alpha c_2+\alpha(2\alpha-\beta\delta)x_1-\alpha\beta x_2}{(\alpha-\beta\delta)(4\alpha-\beta\delta)},$$

$$D_2^{NN*}=\alpha\frac{\beta\delta(\alpha-\beta\delta)+\beta\delta c_1-(2\alpha-\beta\delta)c_2-\alpha\beta\delta x_1+\beta(2\alpha-\beta\delta)x_2}{\beta\delta(\alpha-\beta\delta)(4\alpha-\beta\delta)},$$

$$\pi_{M_1}^{NN*}=\frac{[2\alpha(\alpha-\beta\delta)-(2\alpha-\beta\delta)c_1+\alpha c_2+\alpha(2\alpha-\beta\delta)x_1-\alpha\beta x_2]^2}{(\alpha-\beta\delta)(4\alpha-\beta\delta)^2},$$

$$\pi_{M_2}^{NN*} = \alpha \frac{[\beta\delta(\alpha-\beta\delta)+\beta\delta c_1-(2\alpha-\beta\delta)c_2-\alpha\beta\delta x_1+\beta(2\alpha-\beta\delta)x_2]^2}{\beta\delta(\alpha-\beta\delta)(4\alpha-\beta\delta)^2}.$$

Moreover, $p_1^{NN*} > c_1$, $p_2^{NN*} > c_2$, $D_1^{NN*} > 0$, $D_2^{NN*} > 0$, $\frac{\alpha(p_2-\beta x_2)}{\beta(p_1-\alpha x_1)} < \delta < 1$, $\alpha > \beta\delta$, $\max(0, \frac{-2\alpha(\alpha-\beta\delta)+c_1(2\alpha-\beta\delta)-\alpha c_2+\alpha\beta x_2}{\alpha(2\alpha-\beta\delta)}) < x_1 < \min(1, \frac{\beta\delta(\alpha-\beta\delta)-c_2(2\alpha-\beta\delta)+\beta\delta c_1+\beta(2\alpha-\beta\delta)x_2}{\alpha\beta\delta})$ always hold. That is $\alpha > \beta\delta > c_1$, $\frac{-2\alpha(\alpha-\beta\delta)+c_1(2\alpha-\beta\delta)-\alpha c_2+\alpha\beta x_2}{\alpha(2\alpha-\beta\delta)} < x_1 < \frac{\beta\delta(\alpha-\beta\delta)-c_2(2\alpha-\beta\delta)+\beta\delta c_1+\beta(2\alpha-\beta\delta)x_2}{\alpha\beta\delta}$,

$$\frac{2\alpha(\alpha-\beta\delta)-c_1(2\alpha-\beta\delta)+\alpha c_2}{\alpha\beta} < x_2 < \frac{\beta\beta\delta\delta+c_2(2\alpha-\beta\delta)-\beta\delta c_1}{\beta(2\alpha-\beta\delta)}.$$

Proof of Property 2.

The first derivative of $\pi_{M_1}^{DN}$ w.r.t. p_1 is

$$\frac{\partial \pi_{M_1}^{DN}}{\partial p_1} = \alpha \frac{1-\beta\delta+c_1+x_1-\beta x_2-2p_1+p_2}{1-\beta\delta}. \quad \text{（A3）}$$

The second derivative is $\frac{\partial^2 \pi_{M_1}^{DN}}{\partial p_1^2} = \frac{-2\alpha}{1-\beta\delta} < 0$. Then $\pi_{M_1}^{DN}$ is a concave function w.r.t. p_1.

The first derivative of $\pi_{M_2}^{DN}$ w.r.t. p_2 is

$$\frac{\partial \pi_{M_2}^{DN}}{\partial p_2} = \frac{\beta\delta(1-\alpha)(1-\beta\delta)+(1+\alpha\beta\delta-\beta\delta)c_2-\alpha\beta\delta x_1+\beta(1+\alpha\beta\delta-\beta\delta)x_2+\alpha\beta\delta p_1-2(1+\alpha\beta\delta-\beta\delta)p_2}{\beta\delta(1-\beta\delta)}. \quad \text{（A4）}$$

The second derivative is $\frac{\partial^2 \pi_{M_2}^{DM}}{\partial p_2^2} = \frac{-2(1+\alpha\beta\delta-\beta\delta)}{\beta\delta(1-\beta\delta)} < 0$. Then $\pi_{M_2}^{DN}$ is a concave function w.r.t. p_2.

Proof of Proposition 2.

Let Eq.(A3)=0 and Eq.(A4)=0, then we have

$$p_1^{DN*}=\frac{\begin{array}{l}(1-\beta\delta)(2+\alpha\beta\delta-\beta\delta)+2(1+\alpha\beta\delta-\beta\delta)c_1+(1+\alpha\beta\delta-\beta\delta)c_2\\+(2+\alpha\beta\delta-2\beta\delta)x_1-\beta(1+\alpha\beta\delta-\beta\delta)x_2\end{array}}{4+3\alpha\beta\delta-4\beta\delta},$$

$$p_2^{DN*}=\frac{\begin{array}{l}\beta\delta(2-\alpha)(1-\beta\delta)+\alpha\beta\delta c_1+2(1+\alpha\beta\delta-\beta\delta)c_2\\-\alpha\beta\delta x_1+\beta(2+\alpha\beta\delta-2\beta\delta)x_2\end{array}}{4+3\alpha\beta\delta-4\beta\delta},$$

$$D_1^{DN*}=\alpha\frac{\begin{array}{l}(1-\beta\delta)(2+\alpha\beta\delta-\beta\delta)-(2+\alpha\beta\delta-2\beta\delta)c_1+(1+\alpha\beta\delta-\beta\delta)c_2\\+(2+\alpha\beta\delta-2\beta\delta)x_1-\beta(1+\alpha\beta\delta-\beta\delta)x_2\end{array}}{(1-\beta\delta)(4+3\alpha\beta\delta-4\beta\delta)},$$

$$D_2^{DN*}=(1+\alpha\beta\delta-\beta\delta)\frac{\begin{array}{l}\beta\delta(1-\beta\delta)(2-\alpha)+\alpha\beta\delta c_1-(2+\alpha\beta\delta-2\beta\delta)c_2\\-\alpha\beta\delta x_1+\beta(2+\alpha\beta\delta-2\beta\delta)x_2\end{array}}{\beta\delta(1-\beta\delta)(4+3\alpha\beta\delta-4\beta\delta)},$$

$$\pi_{M_1}^{DN*}=\alpha\frac{\begin{array}{l}[(1-\beta\delta)(2+\alpha\beta\delta-\beta\delta)-(2+\alpha\beta\delta-2\beta\delta)c_1+(1+\alpha\beta\delta-\beta\delta)c_2\\+(2+\alpha\beta\delta-2\beta\delta)x_1-\beta(1+\alpha\beta\delta-\beta\delta)x_2]^2\end{array}}{(1-\beta\delta)(4+3\alpha\beta\delta-4\beta\delta)^2}-C_{d1},$$

$$\pi_{M_2}^{DN*}=\frac{\begin{array}{l}(1+\alpha\beta\delta-\beta\delta)[\beta\delta(2-\alpha)(1-\beta\delta)+\alpha\beta\delta c_1-(2+\alpha\beta\delta-2\beta\delta)c_2\\-\alpha\beta\delta x_1+\beta(2+\alpha\beta\delta-2\beta\delta)x_2]^2\end{array}}{\beta\delta(1-\beta\delta)(4+3\alpha\beta\delta-4\beta\delta)^2}.$$

Moreover, $p_1^{DN*}>c_1$, $p_2^{DN*}>c_2$, $D_1^{DN*}>0$, $D_2^{DN*}>0$, $\frac{p_2-\beta x_2}{\beta(p_1-x_1)}<\delta<1$,

$$\max(0,\frac{\begin{array}{l}c_1(2+\alpha\beta\delta-2\beta\delta)\\-(1-\beta\delta)(2+\alpha\beta\delta-\beta\delta)\\-(1+\alpha\beta\delta-\beta\delta)c_2+\beta(1+\alpha\beta\delta-\beta\delta)x_2\end{array}}{(2+\alpha\beta\delta-2\beta\delta)})<x_1<\min(1,\frac{\begin{array}{l}\beta\delta(2-\alpha)(1-\beta\delta)+\alpha\beta\delta c_1\\-c_2(2+\alpha\beta\delta-2\beta\delta)\\+\beta(2+\alpha\beta\delta-2\beta\delta)x_2\end{array}}{\alpha\beta\delta})$$ will always

hold. That is $$\frac{\begin{array}{l}c_1(2+\alpha\beta\delta-2\beta\delta)\\-(1-\beta\delta)(2+\alpha\beta\delta-\beta\delta)\\-(1+\alpha\beta\delta-\beta\delta)c_2+\beta(1+\alpha\beta\delta-\beta\delta)x_2\end{array}}{(2+\alpha\beta\delta-2\beta\delta)}<x_1<\frac{\begin{array}{l}\beta\delta(2-\alpha)(1-\beta\delta)+\alpha\beta\delta c_1\\-c_2(2+\alpha\beta\delta-2\beta\delta)\\+\beta(2+\alpha\beta\delta-2\beta\delta)x_2\end{array}}{\alpha\beta\delta},$$

$$\frac{\begin{array}{l}(1-\beta\delta)(2+\alpha\beta\delta-\beta\delta)\\-c_1(2+\alpha\beta\delta-2\beta\delta)\\+(1+\alpha\beta\delta-\beta\delta)c_2\end{array}}{\beta(1+\alpha\beta\delta-\beta\delta)}<x_2<\frac{\begin{array}{l}\alpha\beta\delta-\beta\delta(2-\alpha)(1-\beta\delta)\\-\alpha\beta\delta c_1+c_2(2+\alpha\beta\delta-2\beta\delta)\end{array}}{\beta(2+\alpha\beta\delta-2\beta\delta)}.$$

Proof of Property 3.

The first derivative of $\pi_{M_1}^{ND}$ w.r.t. p_1 is

$$\frac{\partial \pi_{M_1}^{ND}}{\partial p_1}=\frac{\begin{array}{c}\alpha(\alpha-\delta)+(\alpha+\beta\delta-\delta)c_1+\alpha(\alpha+\beta\delta-\delta)x_1-\alpha\beta x_2\\ -2(\alpha+\beta\delta-\delta)p_1+\alpha\beta p_2\end{array}}{\alpha(\alpha-\delta)}. \tag{A5}$$

The second derivative is $\frac{\partial^2 \pi_{M_1}^{ND}}{\partial p_1^2}=\frac{-2(\alpha+\beta\delta-\delta)}{\alpha(\alpha-\delta)}<0$. Then $\pi_{M_1}^{ND}$ is a concave function w.r.t. p_1.

The first derivative of $\pi_{M_2}^{ND}$ w.r.t. p_2 is

$$\frac{\partial \pi_{M_2}^{ND}}{\partial p_2}=\beta\frac{\alpha c_2-\alpha\delta x_1+\alpha x_2+\delta p_1-2\alpha p_2}{\delta(\alpha-\delta)}. \tag{A6}$$

The second derivative is $\frac{\partial^2 \pi_{M_2}^{ND}}{\partial p_2^2}=\frac{-2\alpha\beta}{\delta(\alpha-\delta)}<0$. Then $\pi_{M_2}^{ND}$ is a concave function w.r.t. p_2.

Proof of Proposition 3.

Let Eq.(A5)=0 and Eq.(A6)=0, then we have

$$p_1^{ND*}=\frac{\begin{array}{c}2\alpha(\alpha-\delta)+2(\alpha+\beta\delta-\delta)c_1+\alpha\beta c_2\\ +\alpha(2\alpha+\beta\delta-2\delta)x_1-\alpha\beta x_2\end{array}}{4\alpha+3\beta\delta-4\delta},$$

$$p_2^{ND*}=\frac{\begin{array}{c}\alpha\delta(\alpha-\delta)+\delta(\alpha+\beta\delta-\delta)c_1+2\alpha(\alpha+\beta\delta-\delta)c_2\\ -\alpha\delta(\alpha+\beta\delta-\delta)x_1+\alpha(2\alpha+\beta\delta-2\delta)x_2\end{array}}{\alpha(4\alpha+3\beta\delta-4\delta)},$$

$$D_1^{ND*}=(\alpha+\beta\delta-\delta)\frac{\begin{array}{c}2\alpha(\alpha-\delta)-(2\alpha+\beta\delta-2\delta)c_1+\alpha\beta c_2\\ +\alpha(2\alpha+\beta\delta-2\delta)x_1-\alpha\beta x_2\end{array}}{\alpha(\alpha-\delta)(4\alpha+3\beta\delta-4\delta)},$$

$$D_2^{ND*}=\beta\frac{\begin{array}{c}\alpha\delta(\alpha-\delta)+\delta(\alpha+\beta\delta-\delta)c_1-\alpha(2\alpha+\beta\delta-2\delta)c_2\\ -\alpha\delta(\alpha+\beta\delta-\delta)x_1+\alpha(2\alpha+\beta\delta-2\delta)x_2\end{array}}{\delta(\alpha-\delta)(4\alpha+3\beta\delta-4\delta)},$$

$$\pi_{M_1}^{ND*}=\frac{\begin{array}{c}(\alpha+\beta\delta-\delta)[2\alpha(\alpha-\delta)-(2\alpha+\beta\delta-2\delta)c_1+\alpha\beta c_2\\ +\alpha(2\alpha+\beta\delta-2\delta)x_1-\alpha\beta x_2]^2\end{array}}{\alpha(\alpha-\delta)(4\alpha+3\beta\delta-4\delta)^2},$$

$$\pi_{M_2}^{ND*}=\frac{\begin{array}{c}\beta[\alpha\delta(\alpha-\delta)+\delta(\alpha+\beta\delta-\delta)c_1-\alpha(2\alpha+\beta\delta-2\delta)c_2\\ -\alpha\delta(\alpha+\beta\delta-\delta)x_1+\alpha(2\alpha+\beta\delta-2\delta)x_2]^2\end{array}}{\alpha\delta(\alpha-\delta)(4\alpha+3\beta\delta-4\delta)^2}-C_{d2}.$$

Moreover, $p_1^{ND*}>c_1$, $p_2^{ND*}>c_2$, $D_1^{ND*}>0$, $D_2^{ND*}>0$, $\frac{\alpha(p_2-x_2)}{p_1-\alpha x_1}<\delta<1$, $(\alpha-\delta)>0$,

$$\max\left(0,\frac{\begin{array}{l}c_1(2\alpha+\beta\delta-2\delta)\\-2\alpha(\alpha-\delta)\\-\alpha\beta c_2+\alpha\beta x_2\end{array}}{\alpha(2\alpha+\beta\delta-2\delta)}\right)<x_1<\min\left(1,\frac{\begin{array}{l}\alpha\delta(\alpha-\delta)+\delta(\alpha+\beta\delta-\delta)c_1\\-\alpha c_2(2\alpha+\beta\delta-2\delta)\\+\alpha(2\alpha+\beta\delta-2\delta)x_2\end{array}}{\alpha\delta(\alpha+\beta\delta-\delta)}\right)$$ will always hold. That is

$$\alpha>\delta,\quad \frac{\begin{array}{l}c_1(2\alpha+\beta\delta-2\delta)\\-2\alpha(\alpha-\delta)\\-\alpha\beta c_2+\alpha\beta x_2\end{array}}{\alpha(2\alpha+\beta\delta-2\delta)}<x_1<\frac{\begin{array}{l}\alpha\delta(\alpha-\delta)+\delta(\alpha+\beta\delta-\delta)c_1\\-\alpha c_2(2\alpha+\beta\delta-2\delta)\\+\alpha(2\alpha+\beta\delta-2\delta)x_2\end{array}}{\alpha\delta(\alpha+\beta\delta-\delta)},$$

$$\frac{\begin{array}{l}2\alpha(\alpha-\delta)+\alpha\beta c_2\\-c_1(2\alpha+\beta\delta-2\delta)\end{array}}{\alpha\beta}<x_2<\frac{\begin{array}{l}\alpha\beta\delta\delta-\delta(\alpha+\beta\delta-\delta)c_1\\+\alpha c_2(2\alpha+\beta\delta-2\delta)\end{array}}{\alpha(2\alpha+\beta\delta-2\delta)}.$$

Proof of Property 4.

The first derivative of $\pi_{M_1}^{DD}$ w.r.t. p_1 is

$$\frac{\partial\pi_{M_1}^{DD}}{\partial p_1}=\alpha\frac{\begin{array}{l}1-\delta+(1+\beta\delta-\delta)c_1+(1+\beta\delta-\delta)x_1-\beta x_2\\-2(1+\beta\delta-\delta)p_1+\beta p_2\end{array}}{1-\delta}. \qquad \text{(A7)}$$

The second derivative is $\frac{\partial^2\pi_{M_1}^{DD}}{\partial p_1^2}=\frac{-2\alpha(1+\beta\delta-\delta)}{1-\delta}<0$. Then $\pi_{M_1}^{DD}$ is a concave function w.r.t. p_1.

The first derivative of $\pi_{M_2}^{DD}$ w.r.t. p_2 is

$$\frac{\partial\pi_{M_2}^{DD}}{\partial p_2}=\beta\frac{\begin{array}{l}(1-\alpha)\delta(1-\delta)+(1+\alpha\delta-\delta)c_2-\alpha\delta x_1+(1+\alpha\delta-\delta)x_2\\+\alpha\delta p_1-2(1+\alpha\delta-\delta)p_2\end{array}}{\delta(1-\delta)}. \qquad \text{(A8)}$$

The second derivative is $\frac{\partial^2\pi_{M_2}^{DD}}{\partial p_2^2}=\frac{-2\beta(1+\alpha\delta-\delta)}{\delta(1-\delta)}<0$. Then $\pi_{M_2}^{DD}$ is a concave function w.r.t. p_2.

Proof of Proposition 4.

Let Eq.(A7)=0, Eq.(A8)=0, then we have

$$p_1^{DD*}=\frac{\begin{array}{l}(1-\delta)(2+2\alpha\delta-2\delta+\beta\delta-\alpha\beta\delta)\\+2(1+\alpha\delta-\delta)(1+\beta\delta-\delta)c_1+\beta(1+\alpha\delta-\delta)c_2\\+[2(1+\alpha\delta-\delta)(1+\beta\delta-\delta)-\alpha\beta\delta]x_1-\beta(1+\alpha\delta-\delta)x_2\end{array}}{4(1+\alpha\delta-\delta)(1+\beta\delta-\delta)-\alpha\beta\delta},$$

$$p_2^{DD*} = \frac{\begin{array}{l}\delta(1-\delta)(2+2\alpha\delta-2\delta-\alpha+2\beta\delta-2\alpha\beta\delta)\\ +\alpha\delta(1+\beta\delta-\delta)c_1+2(1+\alpha\delta-\delta)(1+\beta\delta-\delta)c_2\\ -\alpha\delta(1+\beta\delta-\delta)x_1+[2(1+\alpha\delta-\delta)(1+\beta\delta-\delta)-\alpha\beta\delta]x_2\end{array}}{4(1+\alpha\delta-\delta)(1+\beta\delta-\delta)-\alpha\beta\delta},$$

$$D_1^{DD*} = \alpha(1+\beta\delta-\delta)\frac{\begin{array}{l}(1-\delta)(2+2\alpha\delta-2\delta+\beta\delta-\alpha\beta\delta)\\ -[2(1+\alpha\delta-\delta)(1+\beta\delta-\delta)-\alpha\beta\delta]c_1+\beta(1+\alpha\delta-\delta)c_2\\ +[2(1+\alpha\delta-\delta)(1+\beta\delta-\delta)-\alpha\beta\delta]x_1-\beta(1+\alpha\delta-\delta)x_2\end{array}}{(1-\delta)[4(1+\alpha\delta-\delta)(1+\beta\delta-\delta)-\alpha\beta\delta]},$$

$$D_2^{DD*} = \beta(1+\alpha\delta-\delta)\frac{\begin{array}{l}\delta(1-\delta)(2+2\beta\delta-2\delta-\alpha-2\alpha\beta\delta+2\alpha\delta)\\ +\alpha\delta(1+\beta\delta-\delta)c_1-[2(1+\alpha\delta-\delta)(1+\beta\delta-\delta)-\alpha\beta\delta]c_2\\ -\alpha\delta(1+\beta\delta-\delta)x_1+[2(1+\alpha\delta-\delta)(1+\beta\delta-\delta)-\alpha\beta\delta]x_2\end{array}}{\delta(1-\delta)[4(1+\alpha\delta-\delta)(1+\beta\delta-\delta)-\alpha\beta\delta]},$$

$$\pi_{M_1}^{DD*} = \alpha(1+\beta\delta-\delta)\frac{\begin{array}{l}\{(1-\delta)(2+2\alpha\delta-2\delta+\beta\delta-\alpha\beta\delta)\\ -[2(1+\alpha\delta-\delta)(1+\beta\delta-\delta)-\alpha\beta\delta]c_1+\beta(1+\alpha\delta-\delta)c_2\\ +[2(1+\alpha\delta-\delta)(1+\beta\delta-\delta)-\alpha\beta\delta]x_1-\beta(1+\alpha\delta-\delta)x_2\}^2\end{array}}{(1-\delta)[4(1+\alpha\delta-\delta)(1+\beta\delta-\delta)-\alpha\beta\delta]^2}-C_{d1},$$

$$\pi_{M_2}^{DD*} = \beta(1+\alpha\delta-\delta)\frac{\begin{array}{l}\{\delta(1-\delta)(2+2\beta\delta-2\delta-\alpha-2\alpha\beta\delta+2\alpha\delta)\\ +\alpha\delta(1+\beta\delta-\delta)c_1-[2(1+\alpha\delta-\delta)(1+\beta\delta-\delta)-\alpha\beta\delta]c_2\\ -\alpha\delta(1+\beta\delta-\delta)x_1+[2(1+\alpha\delta-\delta)(1+\beta\delta-\delta)-\alpha\beta\delta]x_2\}^2\end{array}}{\delta(1-\delta)[4(1+\alpha\delta-\delta)(1+\beta\delta-\delta)-\alpha\beta\delta]^2}-C_{d2}.$$

Moreover, $p_1^{DD*} > c_1$, $p_2^{DD*} > c_2$, $D_1^{DD*} > 0$, $D_2^{DD*} > 0$, $\frac{p_2 - x_2}{p_1 - x_1} < \delta < 1$,

$$\max(0, \frac{\begin{array}{l}c_1[2(1+\alpha\delta-\delta)(1+\beta\delta-\delta)-\alpha\beta\delta]\\ -(1-\delta)(2+2\alpha\delta-2\delta+\beta\delta-\alpha\beta\delta)\\ -\beta(1+\alpha\delta-\delta)c_2+\beta(1+\alpha\delta-\delta)x_2\end{array}}{[2(1+\alpha\delta-\delta)(1+\beta\delta-\delta)-\alpha\beta\delta]}) < x_1 < \max(1, \frac{\begin{array}{l}\delta(1-\delta)(2+2\alpha\delta-2\delta-\alpha+2\beta\delta-2\alpha\beta\delta)\\ +\alpha\delta(1+\beta\delta-\delta)c_1\\ -c_2[2(1+\alpha\delta-\delta)(1+\beta\delta-\delta)-\alpha\beta\delta]\\ +[2(1+\alpha\delta-\delta)(1+\beta\delta-\delta)-\alpha\beta\delta]x_2\end{array}}{\alpha\delta(1+\beta\delta-\delta)})$$

will alwyas hold. That is

$$\frac{\begin{array}{l}c_1[2(1+\alpha\delta-\delta)(1+\beta\delta-\delta)-\alpha\beta\delta]\\ -(1-\delta)(2+2\alpha\delta-2\delta+\beta\delta-\alpha\beta\delta)\\ -\beta(1+\alpha\delta-\delta)c_2+\beta(1+\alpha\delta-\delta)x_2\end{array}}{[2(1+\alpha\delta-\delta)(1+\beta\delta-\delta)-\alpha\beta\delta]} < x_1 < \frac{\begin{array}{l}\delta(1-\delta)(2+2\alpha\delta-2\delta-\alpha+2\beta\delta-2\alpha\beta\delta)\\ +\alpha\delta(1+\beta\delta-\delta)c_1\\ -c_2[2(1+\alpha\delta-\delta)(1+\beta\delta-\delta)-\alpha\beta\delta]\\ +[2(1+\alpha\delta-\delta)(1+\beta\delta-\delta)-\alpha\beta\delta]x_2\end{array}}{\alpha\delta(1+\beta\delta-\delta)},$$

$$\frac{\begin{array}{l}(1-\delta)(2+2\alpha\delta-2\delta+\beta\delta-\alpha\beta\delta)\\+\beta(1+\alpha\delta-\delta)c_2\\-c_1[2(1+\alpha\delta-\delta)(1+\beta\delta-\delta)-\alpha\beta\delta]\end{array}}{\beta(1+\alpha\delta-\delta)}<x_2<\frac{\begin{array}{l}\delta(1+\beta\delta-\delta)(\alpha-2+2\delta)\\+\alpha\delta(1-\delta)(1-2\delta+2\beta\delta)\\-\alpha\delta(1+\beta\delta-\delta)c_1\\+c_2[2(1+\alpha\delta-\delta)(1+\beta\delta-\delta)-\alpha\beta\delta]\end{array}}{[2(1+\alpha\delta-\delta)(1+\beta\delta-\delta)-\alpha\beta\delta]}.$$

Proof of Property 5.

(1) $\frac{\partial p_1^{NN*}}{\partial x_1}=\frac{\alpha(2\alpha-\beta\delta)}{4\alpha-\beta\delta}>0$, $\frac{\partial p_1^{DN*}}{\partial x_1}=\frac{(2+\alpha\beta\delta-2\beta\delta)}{4+3\alpha\beta\delta-4\beta\delta}>0$, $\frac{\partial p_1^{ND*}}{\partial x_1}=\frac{\alpha(2\alpha+\beta\delta-2\delta)x_1}{(4\alpha+3\beta\delta-4\delta)}>0$,

$$\frac{\partial p_1^{DD*}}{\partial x_1}=\frac{2(1+\alpha\delta-\delta)(1+\beta\delta-\delta)-\alpha\beta\delta}{[4(1+\alpha\delta-\delta)(1+\beta\delta-\delta)-\alpha\beta\delta]}>0;$$

$\frac{\partial p_2^{NN*}}{\partial x_1}=\frac{-\alpha\beta\delta}{4\alpha-\beta\delta}<0$, $\frac{\partial p_2^{DN*}}{\partial x_1}=\frac{-\alpha\beta\delta}{4+3\alpha\beta\delta-4\beta\delta}<0$, $\frac{\partial p_2^{ND*}}{\partial x_1}=\frac{-\alpha\delta(\alpha+\beta\delta-\delta)}{\alpha(4\alpha+3\beta\delta-4\delta)}<0$,

$$\frac{\partial p_2^{DD*}}{\partial x_1}=\frac{-\alpha\delta(1+\beta\delta-\delta)}{4(1+\alpha\delta-\delta)(1+\beta\delta-\delta)-\alpha\beta\delta}<0.$$

(2) $\frac{\partial D_1^{NN*}}{\partial x_1}=\frac{\alpha(2\alpha-\beta\delta)}{(\alpha-\beta\delta)(4\alpha-\beta\delta)}>0$, $\frac{\partial D_1^{DN*}}{\partial x_1}=\alpha\frac{(2+\alpha\beta\delta-2\beta\delta)}{(1-\beta\delta)(4+3\alpha\beta\delta-4\beta\delta)}>0$,

$$\frac{\partial D_1^{ND*}}{\partial x_1}=(\alpha+\beta\delta-\delta)\frac{\alpha(2\alpha+\beta\delta-2\delta)}{\alpha(\alpha-\delta)(4\alpha+3\beta\delta-4\delta)}>0,$$

$$\frac{\partial D_2^{DD*}}{\partial x_1}=\frac{-\alpha\beta\delta(1+\beta\delta-\delta)(1+\alpha\delta-\delta)}{\delta(1-\delta)[4(1+\alpha\delta-\delta)(1+\beta\delta-\delta)-\alpha\beta\delta]}<0;$$

$\frac{\partial D_2^{NN*}}{\partial x_1}=\alpha\frac{-\alpha\beta\delta}{\beta\delta(\alpha-\beta\delta)(4\alpha-\beta\delta)}<0$, $\frac{\partial D_2^{DN*}}{\partial x_1}=\frac{-\alpha\beta\delta(1+\alpha\beta\delta-\beta\delta)}{\beta\delta(1-\beta\delta)(4+3\alpha\beta\delta-4\beta\delta)}<0$,

$$\frac{\partial D_2^{ND*}}{\partial x_1}=\beta\frac{-\alpha\delta(\alpha+\beta\delta-\delta)}{\delta(\alpha-\delta)(4\alpha+3\beta\delta-4\delta)}<0,$$

$$\frac{\partial D_1^{DD*}}{\partial x_1}=\alpha(1+\beta\delta-\delta)\frac{[2(1+\alpha\delta-\delta)(1+\beta\delta-\delta)-\alpha\beta\delta]}{(1-\delta)[4(1+\alpha\delta-\delta)(1+\beta\delta-\delta)-\alpha\beta\delta]}>0.$$

(3) $\frac{\partial \pi_{M_1}^{NN*}}{\partial x_1}=2\alpha(2\alpha-\beta\delta)\frac{[2\alpha(\alpha-\beta\delta)-(2\alpha-\beta\delta)c_1+\alpha c_2+\alpha(2\alpha-\beta\delta)x_1-\alpha\beta x_2]}{(\alpha-\beta\delta)(4\alpha-\beta\delta)^2}>0$,

$$\frac{\partial \pi_{M_1}^{DN*}}{\partial x_1} = 2\alpha(2+\alpha\beta\delta-2\beta\delta)\frac{\begin{array}{l}[(1-\beta\delta)(2+\alpha\beta\delta-\beta\delta)\\ -(2+\alpha\beta\delta-2\beta\delta)c_1+(1+\alpha\beta\delta-\beta\delta)c_2\\ +(2+\alpha\beta\delta-2\beta\delta)x_1-\beta(1+\alpha\beta\delta-\beta\delta)x_2]\end{array}}{(1-\beta\delta)(4+3\alpha\beta\delta-4\beta\delta)^2} > 0,$$

$$\frac{\partial \pi_{M_1}^{ND*}}{\partial x_1} = 2\alpha(2\alpha+\beta\delta-2\delta)\frac{\begin{array}{l}(\alpha+\beta\delta-\delta)[2\alpha(\alpha-\delta)-(2\alpha+\beta\delta-2\delta)c_1\\ +\alpha\beta c_2+\alpha(2\alpha+\beta\delta-2\delta)x_1-\alpha\beta x_2]\end{array}}{\alpha(\alpha-\delta)(4\alpha+3\beta\delta-4\delta)^2} > 0,$$

$$\frac{\partial \pi_{M_1}^{DD*}}{\partial x_1} = 2\alpha(1+\beta\delta-\delta)[2(1+\alpha\delta-\delta)(1+\beta\delta-\delta)-\alpha\beta\delta]\frac{\begin{array}{l}\{(1-\delta)(2+2\alpha\delta-2\delta+\beta\delta-\alpha\beta\delta)\\ -[2(1+\alpha\delta-\delta)(1+\beta\delta-\delta)-\alpha\beta\delta]c_1\\ +\beta(1+\alpha\delta-\delta)c_2-\beta(1+\alpha\delta-\delta)x_2\\ +[2(1+\alpha\delta-\delta)(1+\beta\delta-\delta)-\alpha\beta\delta]x_1\}\end{array}}{(1-\delta)[4(1+\alpha\delta-\delta)(1+\beta\delta-\delta)-\alpha\beta\delta]^2} > 0,$$

$$\frac{\partial \pi_{M_2}^{NN*}}{\partial x_1} = -2\alpha^2\beta\delta\frac{[\beta\delta(\alpha-\beta\delta)+\beta\delta c_1-(2\alpha-\beta\delta)c_2-\alpha\beta\delta x_1+\beta(2\alpha-\beta\delta)x_2]}{\beta\delta(\alpha-\beta\delta)(4\alpha-\beta\delta)^2} < 0,$$

$$\frac{\partial \pi_{M_2}^{DN*}}{\partial x_1} = -2\alpha\beta\delta\frac{\begin{array}{l}(1+\alpha\beta\delta-\beta\delta)[\beta\delta(2-\alpha)(1-\beta\delta)+\alpha\beta\delta c_1\\ -(2+\alpha\beta\delta-2\beta\delta)c_2-\alpha\beta\delta x_1+\beta(2+\alpha\beta\delta-2\beta\delta)x_2]\end{array}}{\beta\delta(1-\beta\delta)(4+3\alpha\beta\delta-4\beta\delta)^2} < 0,$$

$$\frac{\partial \pi_{M_2}^{ND*}}{\partial x_1} = -2\alpha\delta(\alpha+\beta\delta-\delta)\frac{\begin{array}{l}\beta[\alpha\delta(\alpha-\delta)+\delta(\alpha+\beta\delta-\delta)c_1-\alpha(2\alpha+\beta\delta-2\delta)c_2\\ -\alpha\delta(\alpha+\beta\delta-\delta)x_1+\alpha(2\alpha+\beta\delta-2\delta)x_2]\end{array}}{\alpha\delta(\alpha-\delta)(4\alpha+3\beta\delta-4\delta)^2} < 0,$$

$$\frac{\partial \pi_{M_2}^{DD*}}{\partial x_1} = -\beta\alpha\delta(1+\alpha\delta-\delta)(1+\beta\delta-\delta)\frac{\begin{array}{l}\{\delta(1-\delta)(2+2\beta\delta-2\delta-\alpha-2\alpha\beta\delta+2\alpha\delta)\\ +\alpha\delta(1+\beta\delta-\delta)c_1--\alpha\delta(1+\beta\delta-\delta)x_1\\ [2(1+\alpha\delta-\delta)(1+\beta\delta-\delta)-\alpha\beta\delta]c_2\\ +[2(1+\alpha\delta-\delta)(1+\beta\delta-\delta)-\alpha\beta\delta]x_2\}\end{array}}{\delta(1-\delta)[4(1+\alpha\delta-\delta)(1+\beta\delta-\delta)-\alpha\beta\delta]^2} < 0.$$

Proof of Property 6.

(1) $\frac{\partial p_1^{NN*}}{\partial x_2} = \frac{-\alpha\beta}{4\alpha-\beta\delta} < 0$, $\frac{\partial p_1^{DN*}}{\partial x_2} = \frac{-\beta(1+\alpha\beta\delta-\beta\delta)}{4+3\alpha\beta\delta-4\beta\delta} < 0$, $\frac{\partial p_1^{ND*}}{\partial x_2} = \frac{-\alpha\beta}{(4\alpha+3\beta\delta-4\delta)} < 0$,

$$\frac{\partial p_1^{DD*}}{\partial x_2} = \frac{-\beta(1+\alpha\delta-\delta)}{[4(1+\alpha\delta-\delta)(1+\beta\delta-\delta)-\alpha\beta\delta]} < 0;$$

$\frac{\partial p_2^{NN*}}{\partial x_2} = \frac{\beta(2\alpha-\beta\delta)}{4\alpha-\beta\delta} > 0$, $\frac{\partial p_2^{DN*}}{\partial x_2} = \frac{\beta(2+\alpha\beta\delta-2\beta\delta)}{4+3\alpha\beta\delta-4\beta\delta} > 0$, $\frac{\partial p_2^{ND*}}{\partial x_2} = \frac{\alpha(2\alpha+\beta\delta-2\delta)}{\alpha(4\alpha+3\beta\delta-4\delta)} > 0$,

$$\frac{\partial p_2^{DD*}}{\partial x_2} = \frac{[2(1+\alpha\delta-\delta)(1+\beta\delta-\delta)-\alpha\beta\delta]}{4(1+\alpha\delta-\delta)(1+\beta\delta-\delta)-\alpha\beta\delta} > 0.$$

(2) $\frac{\partial D_1^{NN*}}{\partial x_2}=\frac{-\alpha\beta}{(\alpha-\beta\delta)(4\alpha-\beta\delta)}<0$, $\frac{\partial D_1^{DN*}}{\partial x_2}=\alpha\frac{-\beta(1+\alpha\beta\delta-\beta\delta)}{(1-\beta\delta)(4+3\alpha\beta\delta-4\beta\delta)}<0$,

$\frac{\partial D_1^{ND*}}{\partial x_2}=\frac{-\alpha\beta(\alpha+\beta\delta-\delta)}{\alpha(\alpha-\delta)(4\alpha+3\beta\delta-4\delta)}<0$, $\frac{\partial D_1^{DD*}}{\partial x_2}=\frac{-\alpha\beta(1+\alpha\delta-\delta)(1+\beta\delta-\delta)}{(1-\delta)[4(1+\alpha\delta-\delta)(1+\beta\delta-\delta)-\alpha\beta\delta]}<0$;

$\frac{\partial D_2^{NN*}}{\partial x_2}=\alpha\frac{\beta(2\alpha-\beta\delta)}{\beta\delta(\alpha-\beta\delta)(4\alpha-\beta\delta)}>0$, $\frac{\partial D_2^{DN*}}{\partial x_2}=\frac{\beta(1+\alpha\beta\delta-\beta\delta)(2+\alpha\beta\delta-2\beta\delta)}{\beta\delta(1-\beta\delta)(4+3\alpha\beta\delta-4\beta\delta)}>0$,

$\frac{\partial D_2^{ND*}}{\partial x_2}=\frac{\alpha\beta(2\alpha+\beta\delta-2\delta)}{\delta(\alpha-\delta)(4\alpha+3\beta\delta-4\delta)}>0$, $\frac{\partial D_2^{DD*}}{\partial x_2}=\frac{\beta(1+\alpha\delta-\delta)[2(1+\alpha\delta-\delta)(1+\beta\delta-\delta)-\alpha\beta\delta]}{\delta(1-\delta)[4(1+\alpha\delta-\delta)(1+\beta\delta-\delta)-\alpha\beta\delta]}>0$.

(3) $\frac{\partial \pi_{M_1}^{NN*}}{\partial x_2}=-2\alpha\beta\frac{[2\alpha(\alpha-\beta\delta)-(2\alpha-\beta\delta)c_1+\alpha c_2+\alpha(2\alpha-\beta\delta)x_1-\alpha\beta x_2]}{(\alpha-\beta\delta)(4\alpha-\beta\delta)^2}<0$,

$$\frac{\partial \pi_{M_1}^{DN*}}{\partial x_2}=-2\alpha\beta(1+\alpha\beta\delta-\beta\delta)\frac{\begin{array}{l}[(1-\beta\delta)(2+\alpha\beta\delta-\beta\delta)\\-(2+\alpha\beta\delta-2\beta\delta)c_1+(1+\alpha\beta\delta-\beta\delta)c_2\\+(2+\alpha\beta\delta-2\beta\delta)x_1-\beta(1+\alpha\beta\delta-\beta\delta)x_2]\end{array}}{(1-\beta\delta)(4+3\alpha\beta\delta-4\beta\delta)^2}<0,$$

$$\frac{\partial \pi_{M_1}^{ND*}}{\partial x_2}=-2\alpha\beta\frac{\begin{array}{l}(\alpha+\beta\delta-\delta)[2\alpha(\alpha-\delta)-(2\alpha+\beta\delta-2\delta)c_1+\alpha\beta c_2\\+\alpha(2\alpha+\beta\delta-2\delta)x_1-\alpha\beta x_2]\end{array}}{\alpha(\alpha-\delta)(4\alpha+3\beta\delta-4\delta)^2}<0,$$

$$\frac{\partial \pi_{M_1}^{DD*}}{\partial x_2}=-2\alpha\beta(1+\alpha\delta-\delta)(1+\beta\delta-\delta)\frac{\begin{array}{l}\{(1-\delta)(2+2\alpha\delta-2\delta+\beta\delta-\alpha\beta\delta)\\-[2(1+\alpha\delta-\delta)(1+\beta\delta-\delta)-\alpha\beta\delta]c_1+\beta(1+\alpha\delta-\delta)c_2\\+[2(1+\alpha\delta-\delta)(1+\beta\delta-\delta)-\alpha\beta\delta]x_1-\beta(1+\alpha\delta-\delta)x_2\}\end{array}}{(1-\delta)[4(1+\alpha\delta-\delta)(1+\beta\delta-\delta)-\alpha\beta\delta]^2}<0;$$

$$\frac{\partial \pi_{M_2}^{NN*}}{\partial x_2}=2\alpha\beta(2\alpha-\beta\delta)\frac{\begin{array}{l}[\beta\delta(\alpha-\beta\delta)+\beta\delta c_1-(2\alpha-\beta\delta)c_2\\-\alpha\beta\delta x_1+\beta(2\alpha-\beta\delta)x_2]\end{array}}{\beta\delta(\alpha-\beta\delta)(4\alpha-\beta\delta)^2}>0,$$

$$\frac{\partial \pi_{M_2}^{DN*}}{\partial x_2}=2\beta(2+\alpha\beta\delta-2\beta\delta)\frac{\begin{array}{l}(1+\alpha\beta\delta-\beta\delta)[\beta\delta(2-\alpha)(1-\beta\delta)+\alpha\beta\delta c_1\\-(2+\alpha\beta\delta-2\beta\delta)c_2-\alpha\beta\delta x_1+\beta(2+\alpha\beta\delta-2\beta\delta)x_2]\end{array}}{\beta\delta(1-\beta\delta)(4+3\alpha\beta\delta-4\beta\delta)^2}>0,$$

$$\frac{\partial \pi_{M_2}^{ND*}}{\partial x_2}=2\alpha(2\alpha+\beta\delta-2\delta)\frac{\begin{array}{l}\beta[\alpha\delta(\alpha-\delta)+\delta(\alpha+\beta\delta-\delta)c_1-\alpha(2\alpha+\beta\delta-2\delta)c_2\\-\alpha\delta(\alpha+\beta\delta-\delta)x_1+\alpha(2\alpha+\beta\delta-2\delta)x_2]\end{array}}{\alpha\delta(\alpha-\delta)(4\alpha+3\beta\delta-4\delta)^2}>0,$$

$$\frac{\partial \pi_{M_2}^{DD*}}{\partial x_2} = 2\beta(1+\alpha\delta-\delta)[2(1+\alpha\delta-\delta)(1+\beta\delta-\delta)-\alpha\beta\delta]\frac{\begin{array}{l}\{\delta(1-\delta)(2+2\beta\delta-2\delta-\alpha-2\alpha\beta\delta+2\alpha\delta)\\ +\alpha\delta(1+\beta\delta-\delta)c_1-\alpha\delta(1+\beta\delta-\delta)x_1\\ -[2(1+\alpha\delta-\delta)(1+\beta\delta-\delta)-\alpha\beta\delta]c_2\\ +[2(1+\alpha\delta-\delta)(1+\beta\delta-\delta)-\alpha\beta\delta]x_2\}\end{array}}{\delta(1-\delta)[4(1+\alpha\delta-\delta)(1+\beta\delta-\delta)-\alpha\beta\delta]^2} > 0.$$

Proof of Property 7.

(1) $$\frac{\partial p_1^{NN*}}{\partial \alpha} = \frac{\begin{array}{l}4\alpha(\alpha-\beta\delta)+2\beta^2\delta^2+4\alpha^2-2\beta\delta c_1-\beta\delta c_2\\ +(8\alpha^2-4\alpha\beta\delta+\beta^2\delta^2)x_1+\beta^2\delta x_2\end{array}}{(4\alpha-\beta\delta)^2}.$$

When $x_1 > \frac{2\beta\delta c_1+\beta\delta c_2-[4\alpha(2\alpha-\beta\delta)+2\beta^2\delta^2]-\beta^2\delta x_2}{(8\alpha^2-4\alpha\beta\delta+\beta^2\delta^2)}$, then $\frac{\partial p_1^{NN*}}{\partial \alpha} > 0$; otherwise, $\frac{\partial p_1^{NN*}}{\partial \alpha} < 0$.

Since $\alpha > \beta\delta > c_1$, $\frac{\begin{array}{l}-2\alpha(\alpha-\beta\delta)+c_1(2\alpha-\beta\delta)\\ -\alpha c_2+\alpha\beta x_2\end{array}}{\alpha(2\alpha-\beta\delta)} < x_1 < \frac{\begin{array}{l}\beta\delta(\alpha-\beta\delta)-c_2(2\alpha-\beta\delta)\\ +\beta\delta c_1+\beta(2\alpha-\beta\delta)x_2\end{array}}{\alpha\beta\delta}$,

$$\frac{2\alpha(\alpha-\beta\delta)-c_1(2\alpha-\beta\delta)+\alpha c_2}{\alpha\beta} < x_2 < \frac{\beta\beta\delta\delta+c_2(2\alpha-\beta\delta)-\beta\delta c_1}{\beta(2\alpha-\beta\delta)},$$

if $\frac{2\beta\delta c_1+\beta\delta c_2-[4\alpha(2\alpha-\beta\delta)+2\beta^2\delta^2]-\beta^2\delta x_2}{(8\alpha^2-4\alpha\beta\delta+\beta^2\delta^2)} < \frac{\begin{array}{l}-2\alpha(\alpha-\beta\delta)+c_1(2\alpha-\beta\delta)\\ -\alpha c_2+\alpha\beta x_2\end{array}}{\alpha(2\alpha-\beta\delta)}$, then $x_1 > \frac{2\beta\delta c_1+\beta\delta c_2-[4\alpha(2\alpha-\beta\delta)+2\beta^2\delta^2]-\beta^2\delta x_2}{(8\alpha^2-4\alpha\beta\delta+\beta^2\delta^2)}$ will always hold. Then $\frac{\partial p_1^{NN*}}{\partial \alpha} > 0$. Moreover,

$$\frac{2\beta\delta c_1+\beta\delta c_2-[4\alpha(2\alpha-\beta\delta)+2\beta^2\delta^2]-\beta^2\delta x_2}{(8\alpha^2-4\alpha\beta\delta+\beta^2\delta^2)} < \frac{\begin{array}{l}-2\alpha(\alpha-\beta\delta)+c_1(2\alpha-\beta\delta)\\ -\alpha c_2+\alpha\beta x_2\end{array}}{\alpha(2\alpha-\beta\delta)},$$

$\frac{\begin{array}{l}-2\alpha\alpha\beta\delta\\ -c_1(2\alpha-\beta\delta)(2\alpha-\beta\delta)\\ +2\alpha\alpha c_2\end{array}}{2\alpha\alpha\beta} < x_2$, $\frac{\begin{array}{l}-2\alpha\alpha\beta\delta\\ -c_1(2\alpha-\beta\delta)(2\alpha-\beta\delta)\\ +2\alpha\alpha c_2\end{array}}{2\alpha\alpha\beta} < \frac{2\alpha(\alpha-\beta\delta)-c_1(2\alpha-\beta\delta)+\alpha c_2}{\alpha\beta}$, then

$\frac{\begin{array}{l}-2\alpha\alpha\beta\delta\\ -c_1(2\alpha-\beta\delta)(2\alpha-\beta\delta)\\ +2\alpha\alpha c_2\end{array}}{2\alpha\alpha\beta} < x_2$ will always hold.

Thus, $\frac{2\beta\delta c_1+\beta\delta c_2-[4\alpha(2\alpha-\beta\delta)+2\beta^2\delta^2]-\beta^2\delta x_2}{(8\alpha^2-4\alpha\beta\delta+\beta^2\delta^2)}<\frac{\begin{array}{c}-2\alpha(\alpha-\beta\delta)+c_1(2\alpha-\beta\delta)\\-\alpha c_2+\alpha\beta x_2\end{array}}{\alpha(2\alpha-\beta\delta)}$,

$x_1>\frac{2\beta\delta c_1+\beta\delta c_2-[4\alpha(2\alpha-\beta\delta)+2\beta^2\delta^2]-\beta^2\delta x_2}{(8\alpha^2-4\alpha\beta\delta+\beta^2\delta^2)}$ will always hold. Then we have $\frac{\partial p_1^{NN*}}{\partial\alpha}>0$.

The following derivation process is consistent with this section, limited to space limitations, we omit the derivation processes.

$\frac{\partial p_1^{DN*}}{\partial\alpha}=\beta\delta(1-\beta\delta)\frac{-(2+\beta\delta)+2c_1+c_2-2x_1-\beta x_2}{(4+3\alpha\beta\delta-4\beta\delta)^2}$. When $x_1<\frac{-(2+\beta\delta)+2c_1+c_2-\beta x_2}{2}$, then $\frac{\partial p_1^{DN*}}{\partial\alpha}>0$; otherwise, $\frac{\partial p_1^{DN*}}{\partial\alpha}<0$. Then

$$\frac{-(2+\beta\delta)+2c_1+c_2-\beta x_2}{2}<\frac{\begin{array}{l}c_1(2+\alpha\beta\delta-2\beta\delta)\\-(1-\beta\delta)(2+\alpha\beta\delta-\beta\delta)\\-(1+\alpha\beta\delta-\beta\delta)c_2+\beta(1+\alpha\beta\delta-\beta\delta)x_2\end{array}}{(2+\alpha\beta\delta-2\beta\delta)}.$$

Thus $x_1<\frac{-(2+\beta\delta)+2c_1+c_2-\beta x_2}{2}$ does not hold. Therefore, $\frac{\partial p_1^{DN*}}{\partial\alpha}<0$.

$$\frac{\partial p_1^{ND*}}{\partial\alpha}=\frac{\begin{array}{l}8(\alpha-\delta)^2+6\beta\delta(2\alpha-\delta)-2\beta\delta c_1-\beta\delta(4-3\beta)c_2\\+x_1[8(\alpha-\delta)^2+2\beta\delta(6\alpha-5\delta)+3\beta^2\delta^2]+\beta\delta(4-3\beta)x_2\end{array}}{(4\alpha+3\beta\delta-4\delta)^2}.$$

When $x_1>\frac{\begin{array}{c}2\beta\delta c_1+\beta\delta(4-3\beta)c_2-8(\alpha-\delta)^2\\-6\beta\delta(2\alpha-\delta)-\beta\delta(4-3\beta)x_2\end{array}}{[8(\alpha-\delta)^2+2\beta\delta(6\alpha-5\delta)+3\beta^2\delta^2]}$, then $\frac{\partial p_1^{ND*}}{\partial\alpha}>0$; otherwise, $\frac{\partial p_1^{ND*}}{\partial\alpha}<0$. Then

$$\frac{\begin{array}{c}2\beta\delta c_1+\beta\delta(4-3\beta)c_2-8(\alpha-\delta)^2\\-6\beta\delta(2\alpha-\delta)-\beta\delta(4-3\beta)x_2\end{array}}{[8(\alpha-\delta)^2+2\beta\delta(6\alpha-5\delta)+3\beta^2\delta^2]}<\frac{\begin{array}{l}c_1(2\alpha+\beta\delta-2\delta)\\-2\alpha(\alpha-\delta)\\-\alpha\beta c_2+\alpha\beta x_2\end{array}}{\alpha(2\alpha+\beta\delta-2\delta)}.$$

Thus $x_1>\frac{\begin{array}{c}2\beta\delta c_1+\beta\delta(4-3\beta)c_2-8(\alpha-\delta)^2\\-6\beta\delta(2\alpha-\delta)-\beta\delta(4-3\beta)x_2\end{array}}{[8(\alpha-\delta)^2+2\beta\delta(6\alpha-5\delta)+3\beta^2\delta^2]}$ will always hold. Therefore, $\frac{\partial p_1^{ND*}}{\partial\alpha}>0$.

$$\frac{\partial p_1^{DD*}}{\partial\alpha}=-\beta\delta(1-\delta)\frac{\beta x_2+2(1+\beta\delta-\delta)x_1-\delta[2(\beta-1)c_1-3\beta+2]-\beta c_2-2c_1+2}{\{\delta[\alpha(4\beta\delta-4\delta-\beta+4)-4(\beta-1)\delta+4\beta-8]+4\}^2}.$$

When $x_1 < \frac{2c_1[1-\delta(1-\beta)]+\beta c_2 -(2+3\beta\delta-2\delta)-\beta x_2}{2(1+\beta\delta-\delta)}$, then $\frac{\partial p_1^{DD*}}{\partial\alpha} > 0$; otherwise, $\frac{\partial p_1^{DD*}}{\partial\alpha} < 0$. Then $\frac{2c_1[1-\delta(1-\beta)]+\beta c_2 -(2+3\beta\delta-2\delta)-\beta x_2}{2(1+\beta\delta-\delta)} < \frac{c_1[2(1+\alpha\delta-\delta)(1+\beta\delta-\delta)-\alpha\beta\delta] -(1-\delta)(2+2\alpha\delta-2\delta+\beta\delta-\alpha\beta\delta) -\beta(1+\alpha\delta-\delta)c_2+\beta(1+\alpha\delta-\delta)x_2}{[2(1+\alpha\delta-\delta)(1+\beta\delta-\delta)-\alpha\beta\delta]}$. Thus $x_1 > \frac{2c_1[1-\delta(1-\beta)]+\beta c_2 -(2+3\beta\delta-2\delta)-\beta x_2}{2(1+\beta\delta-\delta)}$ will always hold. Therefore, $\frac{\partial p_1^{DD*}}{\partial\alpha} < 0$.

(2) $\frac{\partial p_2^{NN*}}{\partial\alpha} = \beta\delta\frac{3\beta\delta-4c_1-2c_2+\beta\delta x_1+2\beta x_2}{(4\alpha-\beta\delta)^2}$. When $x_1 > \frac{4c_1+2c_2-3\beta\delta-2\beta x_2}{\beta\delta}$, then $\frac{\partial p_2^{NN*}}{\partial\alpha} > 0$; otherwise, $\frac{\partial p_2^{NN*}}{\partial\alpha} < 0$. Thus $x_1 > \frac{4c_1+2c_2-3\beta\delta-2\beta x_2}{\beta\delta} > \frac{\beta\delta(\alpha-\beta\delta)-c_2(2\alpha-\beta\delta) +\beta\delta c_1+\beta(2\alpha-\beta\delta)x_2}{\alpha\beta\delta}$ does not hold. Therefore, $\frac{\partial p_2^{NN*}}{\partial\alpha} < 0$.

$\frac{\partial p_2^{DN*}}{\partial\alpha} = 2\beta\delta(1-\beta\delta)\frac{-(2+\beta\delta)+2c_1+c_2-2x_1-\beta x_2}{(4+3\alpha\beta\delta-4\beta\delta)^2}$. When $x_1 < \frac{-(2+\beta\delta)+2c_1+c_2-\beta x_2}{2}$, then $\frac{\partial p_2^{DN*}}{\partial\alpha} > 0$; otherwise, $\frac{\partial p_2^{DN*}}{\partial\alpha} < 0$. Then

$$\frac{-(2+\beta\delta)+2c_1+c_2-\beta x_2}{2} < \frac{c_1(2+\alpha\beta\delta-2\beta\delta) -(1-\beta\delta)(2+\alpha\beta\delta-\beta\delta) -(1+\alpha\beta\delta-\beta\delta)c_2+\beta(1+\alpha\beta\delta-\beta\delta)x_2}{(2+\alpha\beta\delta-2\beta\delta)}.$$

Thus $x_1 < \frac{-(2+\beta\delta)+2c_1+c_2-\beta x_2}{2}$ does not hold. Therefore, $\frac{\partial p_2^{DN*}}{\partial\alpha} < 0$.

$$\frac{\partial p_2^{ND*}}{\partial\alpha} = \delta\frac{3\alpha\alpha\beta\delta-c_1[4\alpha\alpha-\delta(1-\beta)(8\alpha+3\beta\delta-4\delta)] -2\alpha\alpha\beta c_2+\alpha\alpha\beta\delta x_1+2\alpha\alpha\beta x_2}{[\alpha(4\alpha+3\beta\delta-4\delta)]^2}.$$

When $x_1 > \frac{c_1[4\alpha\alpha-\delta(1-\beta)(8\alpha+3\beta\delta-4\delta)] -3\alpha\alpha\beta\delta+2\alpha\alpha\beta c_2-2\alpha\alpha\beta x_2}{\alpha\alpha\beta\delta}$, then $\frac{\partial p_2^{ND*}}{\partial\alpha} > 0$; otherwise, $\frac{\partial p_2^{ND*}}{\partial\alpha} < 0$. Then

$$\frac{\begin{array}{c}c_1[4\alpha\alpha-\delta(1-\beta)(8\alpha+3\beta\delta-4\delta)]\\-3\alpha\alpha\beta\delta+2\alpha\alpha\beta c_2-2\alpha\alpha\beta x_2\end{array}}{\alpha\alpha\beta\delta} > \frac{\begin{array}{c}\alpha\delta(\alpha-\delta)+\delta(\alpha+\beta\delta-\delta)c_1\\-\alpha c_2(2\alpha+\beta\delta-2\delta)\\+\alpha(2\alpha+\beta\delta-2\delta)x_2\end{array}}{\alpha\delta(\alpha+\beta\delta-\delta)}.$$

Thus $x_1 < \frac{\begin{array}{c}c_1[4\alpha\alpha-\delta(1-\beta)(8\alpha+3\beta\delta-4\delta)]\\-3\alpha\alpha\beta\delta+2\alpha\alpha\beta c_2-2\alpha\alpha\beta x_2\end{array}}{\alpha\alpha\beta\delta}$ will always hold. Therefore, $\frac{\partial p_2^{ND*}}{\partial\alpha}<0$.

$\frac{\partial p_2^{DD*}}{\partial\alpha} = -2\delta(1-\delta)(1+\beta\delta-\delta)\frac{\begin{array}{c}\beta x_2+2(1+\beta\delta-\delta)x_1\\-\delta[2(\beta-1)c_1-3\beta+2]\\-\beta c_2-2c_1+2\end{array}}{\begin{array}{c}\{\delta[\alpha(4\beta\delta-4\delta-\beta+4)\\-4(\beta-1)\delta+4\beta-8]+4\}^2\end{array}}$. When $x_1 < \frac{\begin{array}{c}2c_1[1-\delta(1-\beta)]+\beta c_2\\-(2+3\beta\delta-2\delta)-\beta x_2\end{array}}{2(1+\beta\delta-\delta)}$, then $\frac{\partial p_2^{DD*}}{\partial\alpha}>0$; otherwise, $\frac{\partial p_2^{DD*}}{\partial\alpha}<0$. Then $\frac{\begin{array}{c}2c_1[1-\delta(1-\beta)]+\beta c_2\\-(2+3\beta\delta-2\delta)-\beta x_2\end{array}}{2(1+\beta\delta-\delta)} < \frac{\begin{array}{c}c_1[2(1+\alpha\delta-\delta)(1+\beta\delta-\delta)-\alpha\beta\delta]\\-(1-\delta)(2+2\alpha\delta-2\delta+\beta\delta-\alpha\beta\delta)\\-\beta(1+\alpha\delta-\delta)c_2+\beta(1+\alpha\delta-\delta)x_2\end{array}}{[2(1+\alpha\delta-\delta)(1+\beta\delta-\delta)-\alpha\beta\delta]}$.

Thus $x_1 > \frac{\begin{array}{c}2c_1[1-\delta(1-\beta)]+\beta c_2\\-(2+3\beta\delta-2\delta)-\beta x_2\end{array}}{2(1+\beta\delta-\delta)}$ will always hold. Therefore, $\frac{\partial p_2^{DD*}}{\partial\alpha}<0$.

Proof of Property 8.

(1) $\frac{\partial p_1^{NN*}}{\partial\beta} = \alpha\frac{\delta(-6\alpha+2c_1+c_2)-2\alpha\delta x_1-4\alpha x_2}{(4\alpha-\beta\delta)^2}$. When $x_1 < \frac{\delta(-6\alpha+2c_1+c_2)-4\alpha x_2}{2\alpha\delta}$, then $\frac{\partial p_1^{NN*}}{\partial\beta}>0$; otherwise, $\frac{\partial p_1^{NN*}}{\partial\beta}<0$. Then $\frac{\delta(-6\alpha+2c_1+c_2)-4\alpha x_2}{2\alpha\delta} < \frac{\begin{array}{c}-2\alpha(\alpha-\beta\delta)+c_1(2\alpha-\beta\delta)\\-\alpha c_2+\alpha\beta x_2\end{array}}{\alpha(2\alpha-\beta\delta)}$.

Thus $x_1 > \frac{\delta(-6\alpha+2c_1+c_2)-4\alpha x_2}{2\alpha\delta}$ will always hold. Therefore, $\frac{\partial p_1^{NN*}}{\partial\beta}<0$.

$$\frac{\partial p_1^{DN*}}{\partial\beta} = -\frac{\begin{array}{c}(1-\alpha)(4-3\alpha)(x_2+\delta)\beta^2\delta^2-8(1-\alpha)(x_2+\delta)\beta\delta\\+4x_2+\delta[\alpha(2x_1-c_2-2c_1+2)+4]\end{array}}{(4+3\alpha\beta\delta-4\beta\delta)^2}.$$

When $x_1 < \frac{\begin{array}{c}\beta\delta^2(1-\alpha)[8-\beta\delta(4-3\alpha)]-2\delta(\alpha+2)\\+x_2\beta\delta(1-\alpha)[8-(4-3\alpha)\beta\delta]-4x_2\\+\alpha\delta c_2+2\alpha\delta c_1\end{array}}{2\alpha\delta}$, then $\frac{\partial p_1^{DN*}}{\partial\beta}>0$; otherwise, $\frac{\partial p_1^{DN*}}{\partial\beta}<0$. Then

$$\frac{\begin{array}{l}\beta\delta^2(1-\alpha)[8-\beta\delta(4-3\alpha)]-2\delta(\alpha+2)\\ +x_2\beta\delta(1-\alpha)[8-(4-3\alpha)\beta\delta]-4x_2\\ +\alpha\delta c_2+2\alpha\delta c_1\end{array}}{2\alpha\delta}<\frac{\begin{array}{l}c_1(2+\alpha\beta\delta-2\beta\delta)\\ -(1-\beta\delta)(2+\alpha\beta\delta-\beta\delta)\\ -(1+\alpha\beta\delta-\beta\delta)c_2+\beta(1+\alpha\beta\delta-\beta\delta)x_2\end{array}}{(2+\alpha\beta\delta-2\beta\delta)}$$

. Thus

$$x_1>\frac{\begin{array}{l}\beta\delta^2(1-\alpha)[8-\beta\delta(4-3\alpha)]-2\delta(\alpha+2)\\ +x_2\beta\delta(1-\alpha)[8-(4-3\alpha)\beta\delta]-4x_2\\ +\alpha\delta c_2+2\alpha\delta c_1\end{array}}{2\alpha\delta}$$

will always hold. Therefore, $\frac{\partial p_1^{DN*}}{\partial\beta}<0$.

$\frac{\partial p_1^{ND*}}{\partial\beta}=-2(\alpha-\delta)\frac{3\alpha\delta-\delta c_1-2\alpha c_2+\alpha\delta x_1+2\alpha x_2}{(4\alpha+3\beta\delta-4\delta)^2}$. When $x_1<\frac{\delta c_1+2\alpha c_2-3\alpha\delta-2\alpha x_2}{\alpha\delta}$, then $\frac{\partial p_1^{ND*}}{\partial\beta}>0$; otherwise, $\frac{\partial p_1^{ND*}}{\partial\beta}<0$. Then $\frac{\delta c_1+2\alpha c_2-3\alpha\delta-2\alpha x_2}{\alpha\delta}<\frac{\begin{array}{l}c_1(2\alpha+\beta\delta-2\delta)\\ -2\alpha(\alpha-\delta)\\ -\alpha\beta c_2+\alpha\beta x_2\end{array}}{\alpha(2\alpha+\beta\delta-2\delta)}$. Thus $x_1>\frac{\delta c_1+2\alpha c_2-3\alpha\delta-2\alpha x_2}{\alpha\delta}$ will always hold. Therefore, $\frac{\partial p_1^{ND*}}{\partial\beta}<0$.

$$\frac{\partial p_1^{DD*}}{\partial\beta}=-2(1-\delta)(1+\alpha\delta-\delta)\frac{\begin{array}{l}2(1+\alpha\delta-\delta)x_2+\alpha\delta x_1\\ +\delta(2\alpha\delta-2\delta+2+\alpha)\\ -2c_2[1-(1-\alpha)\delta]-\alpha\delta c_1\end{array}}{\begin{array}{l}\{\delta[\beta(4\alpha\delta-4\delta-\alpha+4)\\ -4(\alpha-1)\delta+4\alpha-8]+4\}^2\end{array}}.$$

When $x_1<\frac{\begin{array}{l}2c_2[1-(1-\alpha)\delta]+\alpha\delta c_1\\ -\delta(2\alpha\delta-2\delta+2+\alpha)-2(1+\alpha\delta-\delta)x_2\end{array}}{\alpha\delta}$, then $\frac{\partial p_1^{DD*}}{\partial\beta}>0$; otherwise, $\frac{\partial p_1^{DD*}}{\partial\beta}<0$. Then

$$\frac{\begin{array}{l}2c_2[1-(1-\alpha)\delta]+\alpha\delta c_1\\ -\delta(2\alpha\delta-2\delta+2+\alpha)-2(1+\alpha\delta-\delta)x_2\end{array}}{\alpha\delta}<\frac{\begin{array}{l}c_1[2(1+\alpha\delta-\delta)(1+\beta\delta-\delta)-\alpha\beta\delta]\\ -(1-\delta)(2+2\alpha\delta-2\delta+\beta\delta-\alpha\beta\delta)\\ -\beta(1+\alpha\delta-\delta)c_2+\beta(1+\alpha\delta-\delta)x_2\end{array}}{[2(1+\alpha\delta-\delta)(1+\beta\delta-\delta)-\alpha\beta\delta]}$$

. Thus

$$x_1>\frac{\begin{array}{l}2c_2[1-(1-\alpha)\delta]+\alpha\delta c_1\\ -\delta(2\alpha\delta-2\delta+2+\alpha)-2(1+\alpha\delta-\delta)x_2\end{array}}{\alpha\delta}$$

will always hold. Therefore, $\frac{\partial p_1^{DD*}}{\partial\beta}<0$.

(2) $\frac{\partial p_2^{NN*}}{\partial \beta}=\frac{\delta(4\alpha^2-2\beta\delta+\beta^2\delta^2)+4\alpha\delta c_1+2\alpha\delta c_2-4\alpha^2\delta x_1+(8\alpha^2-8\alpha\beta\delta+\beta^2\delta^2)x_2}{(4\alpha-\beta\delta)^2}$. When $x_1<\frac{\delta(4\alpha^2-2\beta\delta+\beta^2\delta^2)+4\alpha\delta c_1+2\alpha\delta c_2+(8\alpha^2-8\alpha\beta\delta+\beta^2\delta^2)x_2}{4\alpha^2\delta}$, then $\frac{\partial p_2^{NN*}}{\partial \beta}>0$; otherwise, $\frac{\partial p_2^{NN*}}{\partial \beta}<0$. Then $\frac{\delta(4\alpha^2-2\beta\delta+\beta^2\delta^2)+4\alpha\delta c_1+2\alpha\delta c_2+(8\alpha^2-8\alpha\beta\delta+\beta^2\delta^2)x_2}{4\alpha^2\delta}>\frac{\beta\delta(\alpha-\beta\delta)-c_2(2\alpha-\beta\delta)+\beta\delta c_1+\beta(2\alpha-\beta\delta)x_2}{\alpha\beta\delta}$. Thus $x_1<\frac{\delta(4\alpha^2-2\beta\delta+\beta^2\delta^2)+4\alpha\delta c_1+2\alpha\delta c_2+(8\alpha^2-8\alpha\beta\delta+\beta^2\delta^2)x_2}{4\alpha^2\delta}$ will always hold. Therefore, $\frac{\partial p_2^{NN*}}{\partial \beta}>0$.

$\frac{\partial p_2^{DN*}}{\partial \beta}=\frac{x_2[\beta^2\delta^2(2-\alpha)(4-3\alpha)-8\beta\delta(2-\alpha)+8]+\delta(2-\alpha)[\beta^2\delta^2(4-3\alpha)-8\beta\delta+4]-4\alpha\delta x_1+2\alpha\delta c_2+4\alpha\delta c_1}{(4+3\alpha\beta\delta-4\beta\delta)^2}$. When $x_1<\frac{x_2[\beta^2\delta^2(2-\alpha)(4-3\alpha)-8\beta\delta(2-\alpha)+8]+\delta(2-\alpha)[\beta^2\delta^2(4-3\alpha)-8\beta\delta+4]+2\alpha\delta c_2+4\alpha\delta c_1}{4\alpha\delta}$, then $\frac{\partial p_2^{DN*}}{\partial \beta}>0$; otherwise, $\frac{\partial p_2^{DN*}}{\partial \beta}<0$.

Then $\frac{x_2[\beta^2\delta^2(2-\alpha)(4-3\alpha)-8\beta\delta(2-\alpha)+8]+\delta(2-\alpha)[\beta^2\delta^2(4-3\alpha)-8\beta\delta+4]+2\alpha\delta c_2+4\alpha\delta c_1}{4\alpha\delta}>\frac{\beta\delta(2-\alpha)(1-\beta\delta)+\alpha\beta\delta c_1-c_2(2+\alpha\beta\delta-2\beta\delta)+\beta(2+\alpha\beta\delta-2\beta\delta)x_2}{\alpha\beta\delta}$.

Thus $x_1<\frac{x_2[\beta^2\delta^2(2-\alpha)(4-3\alpha)-8\beta\delta(2-\alpha)+8]+\delta(2-\alpha)[\beta^2\delta^2(4-3\alpha)-8\beta\delta+4]+2\alpha\delta c_2+4\alpha\delta c_1}{4\alpha\delta}$ will always hold. Therefore, $\frac{\partial p_2^{DN*}}{\partial \beta}>0$.

$\frac{\partial p_2^{ND*}}{\partial \beta}=\frac{-\delta(\alpha-\delta)(3\alpha\delta+\alpha\delta x_1+2\alpha x_2-\delta c_1-2\alpha c_2)}{\alpha(4\alpha+3\beta\delta-4\delta)^2}$. When $x_1<\frac{\delta c_1+2\alpha c_2-3\alpha\delta-2\alpha x_2}{\alpha\delta}$, then $\frac{\partial p_2^{ND*}}{\partial \beta}>0$; otherwise, $\frac{\partial p_2^{ND*}}{\partial \beta}<0$. Then $\frac{\delta c_1+2\alpha c_2-3\alpha\delta-2\alpha x_2}{\alpha\delta}<\frac{c_1(2\alpha+\beta\delta-2\delta)-2\alpha(\alpha-\delta)-\alpha\beta c_2+\alpha\beta x_2}{\alpha(2\alpha+\beta\delta-2\delta)}$. Thus $x_1>\frac{\delta c_1+2\alpha c_2-3\alpha\delta-2\alpha x_2}{\alpha\delta}$ will always hold. Therefore, $\frac{\partial p_2^{ND*}}{\partial \beta}<0$.

$$\frac{\partial p_2^{DD*}}{\partial \beta}=-\alpha\delta(1-\delta)\frac{\begin{array}{l}2(1+\alpha\delta-\delta)x_2+\alpha\delta x_1\\-2c_2[1-\delta(1-\alpha)]-\alpha\delta c_1\\+\delta(2\alpha\delta-2\delta+\alpha+2)\end{array}}{\begin{array}{l}\{\delta[\beta(4\alpha\delta-4\delta-\alpha+4)\\-4(\alpha-1)\delta+4\alpha-8]+4\}^2\end{array}}$$

. When $x_1<\frac{\begin{array}{c}2c_2[1-\delta(1-\alpha)]+\alpha\delta c_1\\-\delta(2\alpha\delta-2\delta+\alpha+2)-2(1+\alpha\delta-\delta)x_2\end{array}}{\alpha\delta}$, then $\frac{\partial p_2^{DD*}}{\partial \beta}>0$; otherwise, $\frac{\partial p_2^{DD*}}{\partial \beta}<0$. Then

$$\frac{\begin{array}{l}2c_2[1-(1-\alpha)\delta]+\alpha\delta c_1\\-\delta(2\alpha\delta-2\delta+2+\alpha)-2(1+\alpha\delta-\delta)x_2\end{array}}{\alpha\delta}<\frac{\begin{array}{l}c_1[2(1+\alpha\delta-\delta)(1+\beta\delta-\delta)-\alpha\beta\delta]\\-(1-\delta)(2+2\alpha\delta-2\delta+\beta\delta-\alpha\beta\delta)\\-\beta(1+\alpha\delta-\delta)c_2+\beta(1+\alpha\delta-\delta)x_2\end{array}}{[2(1+\alpha\delta-\delta)(1+\beta\delta-\delta)-\alpha\beta\delta]}$$

. Thus $x_1>\frac{\begin{array}{l}2c_2[1-(1-\alpha)\delta]+\alpha\delta c_1\\-\delta(2\alpha\delta-2\delta+2+\alpha)-2(1+\alpha\delta-\delta)x_2\end{array}}{\alpha\delta}$ will always hold. Therefore, $\frac{\partial p_2^{DD*}}{\partial \beta}<0$.

Proof of Proposition 5.

When $\alpha>\beta\delta>c_1$, $\alpha>\delta$,

$$\max\left(\begin{array}{l}\frac{\begin{array}{l}-2\alpha(\alpha-\beta\delta)+c_1(2\alpha-\beta\delta)\\-\alpha c_2+\alpha\beta x_2\end{array}}{\alpha(2\alpha-\beta\delta)},\frac{\begin{array}{l}c_1(2+\alpha\beta\delta-2\beta\delta)\\-(1-\beta\delta)(2+\alpha\beta\delta-\beta\delta)\\-(1+\alpha\beta\delta-\beta\delta)c_2+\beta(1+\alpha\beta\delta-\beta\delta)x_2\end{array}}{(2+\alpha\beta\delta-2\beta\delta)}\\,\frac{\begin{array}{l}c_1(2\alpha+\beta\delta-2\delta)\\-2\alpha(\alpha-\delta)\\-\alpha\beta c_2+\alpha\beta x_2\end{array}}{\alpha(2\alpha+\beta\delta-2\delta)},\frac{\begin{array}{l}c_1[2(1+\alpha\delta-\delta)(1+\beta\delta-\delta)-\alpha\beta\delta]\\-(1-\delta)(2+2\alpha\delta-2\delta+\beta\delta-\alpha\beta\delta)\\-\beta(1+\alpha\delta-\delta)c_2+\beta(1+\alpha\delta-\delta)x_2\end{array}}{[2(1+\alpha\delta-\delta)(1+\beta\delta-\delta)-\alpha\beta\delta]}\end{array}\right)<x_1$$

$$<\min\left(\begin{array}{l}\frac{\begin{array}{l}\beta\delta(\alpha-\beta\delta)-c_2(2\alpha-\beta\delta)\\+\beta\delta c_1+\beta(2\alpha-\beta\delta)x_2\end{array}}{\alpha\beta\delta},\frac{\begin{array}{l}\beta\delta(2-\alpha)(1-\beta\delta)+\alpha\beta\delta c_1\\-c_2(2+\alpha\beta\delta-2\beta\delta)\\+\beta(2+\alpha\beta\delta-2\beta\delta)x_2\end{array}}{\alpha\beta\delta},\\ \frac{\begin{array}{l}\alpha\delta(\alpha-\delta)+\delta(\alpha+\beta\delta-\delta)c_1\\-\alpha c_2(2\alpha+\beta\delta-2\delta)\\+\alpha(2\alpha+\beta\delta-2\delta)x_2\end{array}}{\alpha\delta(\alpha+\beta\delta-\delta)},\frac{\begin{array}{l}\delta(1-\delta)(2+2\alpha\delta-2\delta-\alpha+2\beta\delta-2\alpha\beta\delta)\\+\alpha\delta(1+\beta\delta-\delta)c_1\\-c_2[2(1+\alpha\delta-\delta)(1+\beta\delta-\delta)-\alpha\beta\delta]\\+[2(1+\alpha\delta-\delta)(1+\beta\delta-\delta)-\alpha\beta\delta]x_2\end{array}}{\alpha\delta(1+\beta\delta-\delta)}\end{array}\right),$$

$$\max\left(\begin{array}{l}\dfrac{2\alpha(\alpha-\beta\delta)-c_1(2\alpha-\beta\delta)+\alpha c_2}{\alpha\beta},\dfrac{\begin{array}{l}(1-\beta\delta)(2+\alpha\beta\delta-\beta\delta)\\-c_1(2+\alpha\beta\delta-2\beta\delta)\\+(1+\alpha\beta\delta-\beta\delta)c_2\end{array}}{\beta(1+\alpha\beta\delta-\beta\delta)},\\ \dfrac{\begin{array}{l}2\alpha(\alpha-\delta)+\alpha\beta c_2\\-c_1(2\alpha+\beta\delta-2\delta)\end{array}}{\alpha\beta},\dfrac{\begin{array}{l}(1-\delta)(2+2\alpha\delta-2\delta+\beta\delta-\alpha\beta\delta)\\+\beta(1+\alpha\delta-\delta)c_2\\-c_1[2(1+\alpha\delta-\delta)(1+\beta\delta-\delta)-\alpha\beta\delta]\end{array}}{\beta(1+\alpha\delta-\delta)}\end{array}\right)<x_2$$

$$<\min\left(\begin{array}{l}\dfrac{\beta\beta\delta\delta+c_2(2\alpha-\beta\delta)-\beta\delta c_1}{\beta(2\alpha-\beta\delta)},\dfrac{\begin{array}{l}\alpha\beta\delta-\beta\delta(2-\alpha)(1-\beta\delta)\\-\alpha\beta\delta c_1+c_2(2+\alpha\beta\delta-2\beta\delta)\end{array}}{\beta(2+\alpha\beta\delta-2\beta\delta)},\\ \dfrac{\begin{array}{l}\alpha\beta\delta\delta-\delta(\alpha+\beta\delta-\delta)c_1\\+\alpha c_2(2\alpha+\beta\delta-2\delta)\end{array}}{\alpha(2\alpha+\beta\delta-2\delta)},\dfrac{\begin{array}{l}\delta(1+\beta\delta-\delta)(\alpha-2+2\delta)\\+\alpha\delta(1-\delta)(1-2\delta+2\beta\delta)\\-\alpha\delta(1+\beta\delta-\delta)c_1\\+c_2[2(1+\alpha\delta-\delta)(1+\beta\delta-\delta)-\alpha\beta\delta]\end{array}}{[2(1+\alpha\delta-\delta)(1+\beta\delta-\delta)-\alpha\beta\delta]}\end{array}\right)$$

, these four situations (NN,ND,DN,DD) may happen simultaneously. That is, $\alpha>\beta\delta>c_1$, $\alpha>\delta$,

$$\frac{\begin{array}{l}c_1[2(1+\alpha\delta-\delta)(1+\beta\delta-\delta)-\alpha\beta\delta]\\-(1-\delta)(2+2\alpha\delta-2\delta+\beta\delta-\alpha\beta\delta)\\-\beta(1+\alpha\delta-\delta)c_2+\beta(1+\alpha\delta-\delta)x_2\end{array}}{[2(1+\alpha\delta-\delta)(1+\beta\delta-\delta)-\alpha\beta\delta]}<x_1<\frac{\begin{array}{l}\delta(1-\delta)(2+2\alpha\delta-2\delta-\alpha+2\beta\delta-2\alpha\beta\delta)\\+\alpha\delta(1+\beta\delta-\delta)c_1\\-c_2[2(1+\alpha\delta-\delta)(1+\beta\delta-\delta)-\alpha\beta\delta]\\+[2(1+\alpha\delta-\delta)(1+\beta\delta-\delta)-\alpha\beta\delta]x_2\end{array}}{\alpha\delta(1+\beta\delta-\delta)},$$

$$\frac{\begin{array}{l}(1-\delta)(2+2\alpha\delta-2\delta+\beta\delta-\alpha\beta\delta)\\+\beta(1+\alpha\delta-\delta)c_2\\-c_1[2(1+\alpha\delta-\delta)(1+\beta\delta-\delta)-\alpha\beta\delta]\end{array}}{\beta(1+\alpha\delta-\delta)}<x_2<\frac{\begin{array}{l}\delta(1+\beta\delta-\delta)(\alpha-2+2\delta)\\+\alpha\delta(1-\delta)(1-2\delta+2\beta\delta)\\-\alpha\delta(1+\beta\delta-\delta)c_1\\+c_2[2(1+\alpha\delta-\delta)(1+\beta\delta-\delta)-\alpha\beta\delta]\end{array}}{[2(1+\alpha\delta-\delta)(1+\beta\delta-\delta)-\alpha\beta\delta]}$$

. Then we have the following analysis.

$$p_1^{NN*}-p_1^{DN*}=\frac{2\alpha(\alpha-\beta\delta)+2\alpha c_1+\alpha c_2+\alpha(2\alpha-\beta\delta)x_1-\alpha\beta x_2}{4\alpha-\beta\delta}$$

（1） $$-\frac{\begin{array}{l}(1-\beta\delta)(2+\alpha\beta\delta-\beta\delta)+2(1+\alpha\beta\delta-\beta\delta)c_1+(1+\alpha\beta\delta-\beta\delta)c_2\\+(2+\alpha\beta\delta-2\beta\delta)x_1-\beta(1+\alpha\beta\delta-\beta\delta)x_2\end{array}}{4+3\alpha\beta\delta-4\beta\delta}.$$

When $x_1<-1+\dfrac{\beta\delta(1+\alpha-\beta\delta)(2c_1+c_2-\beta x_2-\beta\delta)}{(4\alpha-\beta\delta)(2+\alpha\beta\delta-2\beta\delta)+2\alpha\beta\delta(1+\alpha-\beta\delta)}$, then $p_1^{NN*}>p_1^{DN*}$; otherwise

$p_1^{NN*} < p_1^{DN*}$. Thus $-1+\frac{\beta\delta(1+\alpha-\beta\delta)(2c_1+c_2-\beta x_2-\beta\delta)}{(4\alpha-\beta\delta)(2+\alpha\beta\delta-2\beta\delta)+2\alpha\beta\delta(1+\alpha-\beta\delta)}<0$,

$x_1<-1+\frac{\beta\delta(1+\alpha-\beta\delta)(2c_1+c_2-\beta x_2-\beta\delta)}{(4\alpha-\beta\delta)(2+\alpha\beta\delta-2\beta\delta)+2\alpha\beta\delta(1+\alpha-\beta\delta)}$ do not hold. Therefore, $p_1^{NN*} < p_1^{DN*}$.

$$p_1^{NN*}-p_1^{ND*}=\frac{2\alpha(\alpha-\beta\delta)+2\alpha c_1+\alpha c_2+\alpha(2\alpha-\beta\delta)x_1-\alpha\beta x_2}{4\alpha-\beta\delta}$$
$$-\frac{2\alpha(\alpha-\delta)+2(\alpha+\beta\delta-\delta)c_1+\alpha\beta c_2+\alpha(2\alpha+\beta\delta-2\delta)x_1-\alpha\beta x_2}{(4\alpha+3\beta\delta-4\delta)}.$$

Since $x_1>0>\frac{-2\beta\delta\delta(3\alpha-c_1)-\alpha c_2[4(\alpha-\delta)-\beta\delta]-4\alpha\beta\delta x_2}{2\alpha\beta\delta\delta}$, then $p_1^{NN*}>p_1^{ND*}$.

$$p_1^{DN*}-p_1^{DD*}=\frac{\begin{array}{l}(1-\beta\delta)(2+\alpha\beta\delta-\beta\delta)+2(1+\alpha\beta\delta-\beta\delta)c_1+(1+\alpha\beta\delta-\beta\delta)c_2\\+(2+\alpha\beta\delta-2\beta\delta)x_1-\beta(1+\alpha\beta\delta-\beta\delta)x_2\end{array}}{4+3\alpha\beta\delta-4\beta\delta}$$
$$-\frac{\begin{array}{l}(1-\delta)(2+2\alpha\delta-2\delta+\beta\delta-\alpha\beta\delta)\\+2(1+\alpha\delta-\delta)(1+\beta\delta-\delta)c_1+\beta(1+\alpha\delta-\delta)c_2\\+[2(1+\alpha\delta-\delta)(1+\beta\delta-\delta)-\alpha\beta\delta]x_1-\beta(1+\alpha\delta-\delta)x_2\end{array}}{[4(1+\alpha\delta-\delta)(1+\beta\delta-\delta)-\alpha\beta\delta]}.$$

When $x_1>\frac{\begin{array}{l}(1-\beta\delta)(2+\alpha\beta\delta-\beta\delta)[4(1+\alpha\delta-\delta)(1+\beta\delta-\delta)-\alpha\beta\delta]\\-(1-\delta)(2+2\alpha\delta-2\delta+\beta\delta-\alpha\beta\delta)(4+3\alpha\beta\delta-4\beta\delta)\\-2\alpha\beta\delta\delta c_1(1-\beta)(2-\alpha-\delta+\alpha\delta)\\+c_2(1-\beta)[4(1+\alpha\delta-\delta)(1-\delta)(1+\alpha\beta\delta-\beta\delta)-\alpha\beta\delta]\\-\beta x_2(1+\alpha\delta-\delta)(\beta\delta-\delta)(4+3\alpha\beta\delta-4\beta\delta)\\+\alpha\beta\delta\beta x_2[2+\alpha\delta+\alpha\beta\delta-2\delta+(\alpha\delta-\delta)(\beta\delta-\delta)\end{array}}{-2\alpha\beta\delta\delta(1-\beta)(2-\alpha-\delta+\alpha\delta)}$, then $p_1^{DN*}>p_1^{DD*}$; otherwise $p_1^{DN*}<p_1^{DD*}$.

Thus $\frac{\begin{array}{l}(1-\beta\delta)(2+\alpha\beta\delta-\beta\delta)[4(1+\alpha\delta-\delta)(1+\beta\delta-\delta)-\alpha\beta\delta]\\-(1-\delta)(2+2\alpha\delta-2\delta+\beta\delta-\alpha\beta\delta)(4+3\alpha\beta\delta-4\beta\delta)\\-2\alpha\beta\delta\delta c_1(1-\beta)(2-\alpha-\delta+\alpha\delta)\\+c_2(1-\beta)[4(1+\alpha\delta-\delta)(1-\delta)(1+\alpha\beta\delta-\beta\delta)-\alpha\beta\delta]\\-\beta x_2(1+\alpha\delta-\delta)(\beta\delta-\delta)(4+3\alpha\beta\delta-4\beta\delta)\\+\alpha\beta\delta\beta x_2[2+\alpha\delta+\alpha\beta\delta-2\delta+(\alpha\delta-\delta)(\beta\delta-\delta)\end{array}}{-2\alpha\beta\delta\delta(1-\beta)(2-\alpha-\delta+\alpha\delta)}<\frac{\begin{array}{l}c_1[2(1+\alpha\delta-\delta)(1+\beta\delta-\delta)-\alpha\beta\delta]\\-(1-\delta)(2+2\alpha\delta-2\delta+\beta\delta-\alpha\beta\delta)\\-\beta(1+\alpha\delta-\delta)c_2+\beta(1+\alpha\delta-\delta)x_2\end{array}}{[2(1+\alpha\delta-\delta)(1+\beta\delta-\delta)-\alpha\beta\delta]}$.

Therefore, when these four situations happen simultaneously,

then $x_1 > \dfrac{\begin{array}{l}(1-\beta\delta)(2+\alpha\beta\delta-\beta\delta)[4(1+\alpha\delta-\delta)(1+\beta\delta-\delta)-\alpha\beta\delta] \\ -(1-\delta)(2+2\alpha\delta-2\delta+\beta\delta-\alpha\beta\delta)(4+3\alpha\beta\delta-4\beta\delta) \\ -2\alpha\beta\delta\delta c_1(1-\beta)(2-\alpha-\delta+\alpha\delta) \\ +c_2(1-\beta)[4(1+\alpha\delta-\delta)(1-\delta)(1+\alpha\beta\delta-\beta\delta)-\alpha\beta\delta] \\ -\beta x_2(1+\alpha\delta-\delta)(\beta\delta-\delta)(4+3\alpha\beta\delta-4\beta\delta) \\ +\alpha\beta\delta\beta x_2[2+\alpha\delta+\alpha\beta\delta-2\delta+(\alpha\delta-\delta)(\beta\delta-\delta)\end{array}}{-2\alpha\beta\delta\delta(1-\beta)(2-\alpha-\delta+\alpha\delta)}$ will always hold. Therefore, $p_1^{DN*} > p_1^{DD*}$.

$$p_1^{NN*} - p_1^{DD*} = \frac{2\alpha(\alpha-\beta\delta)+2\alpha c_1+\alpha c_2+\alpha(2\alpha-\beta\delta)x_1-\alpha\beta x_2}{4\alpha-\beta\delta} - \frac{\begin{array}{l}(1-\delta)(2+2\alpha\delta-2\delta+\beta\delta-\alpha\beta\delta) \\ +2(1+\alpha\delta-\delta)(1+\beta\delta-\delta)c_1+\beta(1+\alpha\delta-\delta)c_2 \\ +[2(1+\alpha\delta-\delta)(1+\beta\delta-\delta)-\alpha\beta\delta]x_1-\beta(1+\alpha\delta-\delta)x_2\end{array}}{[4(1+\alpha\delta-\delta)(1+\beta\delta-\delta)-\alpha\beta\delta]}.$$

When $x_1 < \dfrac{\begin{array}{l}2\alpha(\alpha-\beta\delta)[4(1+\alpha\delta-\delta)(1+\beta\delta-\delta)-\alpha\beta\delta] \\ -(1-\delta)(2+2\alpha\delta-2\delta+\beta\delta-\alpha\beta\delta)(4\alpha-\beta\delta) \\ +2\alpha c_1[4(1+\alpha\delta-\delta)(1+\beta\delta-\delta)-\alpha\beta\delta] \\ -2(1+\alpha\delta-\delta)(1+\beta\delta-\delta)(4\alpha-\beta\delta)c_1 \\ +\alpha c_2[4(1+\alpha\delta-\delta)(1+\beta\delta-\delta)-\alpha\beta\delta] \\ -\beta(1+\alpha\delta-\delta)(4\alpha-\beta\delta)c_2 \\ -\alpha\beta x_2[4(1+\alpha\delta-\delta)(1+\beta\delta-\delta)-\alpha\beta\delta] \\ +\beta(1+\alpha\delta-\delta)(4\alpha-\beta\delta)x_2\end{array}}{\begin{array}{l}(1-\alpha)[2(1+\alpha\delta-\delta)(1+\beta\delta-\delta)(4\alpha-\beta\delta) \\ +\alpha\beta\delta(2-2\delta-2\alpha+\beta\delta)]\end{array}}$, then $p_1^{NN*} > p_1^{DD*}$; otherwise $p_1^{NN*} < p_1^{DD*}$.

Since $\dfrac{\begin{array}{l}2\alpha(\alpha-\beta\delta)[4(1+\alpha\delta-\delta)(1+\beta\delta-\delta)-\alpha\beta\delta] \\ -(1-\delta)(2+2\alpha\delta-2\delta+\beta\delta-\alpha\beta\delta)(4\alpha-\beta\delta) \\ +2\alpha c_1[4(1+\alpha\delta-\delta)(1+\beta\delta-\delta)-\alpha\beta\delta] \\ -2(1+\alpha\delta-\delta)(1+\beta\delta-\delta)(4\alpha-\beta\delta)c_1 \\ +\alpha c_2[4(1+\alpha\delta-\delta)(1+\beta\delta-\delta)-\alpha\beta\delta] \\ -\beta(1+\alpha\delta-\delta)(4\alpha-\beta\delta)c_2 \\ -\alpha\beta x_2[4(1+\alpha\delta-\delta)(1+\beta\delta-\delta)-\alpha\beta\delta] \\ +\beta(1+\alpha\delta-\delta)(4\alpha-\beta\delta)x_2\end{array}}{\begin{array}{l}(1-\alpha)[2(1+\alpha\delta-\delta)(1+\beta\delta-\delta)(4\alpha-\beta\delta) \\ +\alpha\beta\delta(2-2\delta-2\alpha+\beta\delta)]\end{array}} < \dfrac{\begin{array}{l}c_1[2(1+\alpha\delta-\delta)(1+\beta\delta-\delta)-\alpha\beta\delta] \\ -(1-\delta)(2+2\alpha\delta-2\delta+\beta\delta-\alpha\beta\delta) \\ -\beta(1+\alpha\delta-\delta)c_2+\beta(1+\alpha\delta-\delta)x_2\end{array}}{[2(1+\alpha\delta-\delta)(1+\beta\delta-\delta)-\alpha\beta\delta]}$, when these four situations happen simultaneously, then

$$x_1 > \frac{\begin{array}{l} 2\alpha(\alpha-\beta\delta)[4(1+\alpha\delta-\delta)(1+\beta\delta-\delta)-\alpha\beta\delta] \\ -(1-\delta)(2+2\alpha\delta-2\delta+\beta\delta-\alpha\beta\delta)(4\alpha-\beta\delta) \\ +2\alpha c_1[4(1+\alpha\delta-\delta)(1+\beta\delta-\delta)-\alpha\beta\delta] \\ -2(1+\alpha\delta-\delta)(1+\beta\delta-\delta)(4\alpha-\beta\delta)c_1 \\ +\alpha c_2[4(1+\alpha\delta-\delta)(1+\beta\delta-\delta)-\alpha\beta\delta] \\ -\beta(1+\alpha\delta-\delta)(4\alpha-\beta\delta)c_2 \\ -\alpha\beta x_2[4(1+\alpha\delta-\delta)(1+\beta\delta-\delta)-\alpha\beta\delta] \\ +\beta(1+\alpha\delta-\delta)(4\alpha-\beta\delta)x_2 \end{array}}{\begin{array}{l}(1-\alpha)[2(1+\alpha\delta-\delta)(1+\beta\delta-\delta)(4\alpha-\beta\delta) \\ +\alpha\beta\delta(2-2\delta-2\alpha+\beta\delta)]\end{array}}$$

will always hold. Therefore, $p_1^{NN*} < p_1^{DD*}$.

(2) $$p_2^{NN*} - p_2^{DN*} = \frac{\begin{array}{c}\beta\delta(\alpha-\beta\delta)+\beta\delta c_1+2\alpha c_2 \\ -\alpha\beta\delta x_1+\beta(2\alpha-\beta\delta)x_2\end{array}}{4\alpha-\beta\delta} - \frac{\begin{array}{c}\beta\delta(2-\alpha)(1-\beta\delta)+\alpha\beta\delta c_1+2(1+\alpha\beta\delta-\beta\delta)c_2 \\ -\alpha\beta\delta x_1+\beta(2+\alpha\beta\delta-2\beta\delta)x_2\end{array}}{4+3\alpha\beta\delta-4\beta\delta}.$$

When $x_1 < \frac{\begin{array}{c}-2(1-\beta\delta)(2\alpha+\beta\delta)-3\alpha\beta\delta \\ +4c_1(1+\alpha-\beta\delta)+2c_2(1+\alpha-\beta\delta) \\ -2\beta x_2(1+\alpha-\beta\delta)\end{array}}{\alpha(4-3\beta\delta)}$, then $p_2^{NN*} > p_2^{DN*}$; otherwise $p_2^{NN*} < p_2^{DN*}$.

Since $\frac{\begin{array}{c}-2(1-\beta\delta)(2\alpha+\beta\delta)-3\alpha\beta\delta \\ +4c_1(1+\alpha-\beta\delta)+2c_2(1+\alpha-\beta\delta) \\ -2\beta x_2(1+\alpha-\beta\delta)\end{array}}{\alpha(4-3\beta\delta)} > \frac{\begin{array}{l}\delta(1-\delta)(2+2\alpha\delta-2\delta-\alpha+2\beta\delta-2\alpha\beta\delta) \\ +\alpha\delta(1+\beta\delta-\delta)c_1 \\ -c_2[2(1+\alpha\delta-\delta)(1+\beta\delta-\delta)-\alpha\beta\delta] \\ +[2(1+\alpha\delta-\delta)(1+\beta\delta-\delta)-\alpha\beta\delta]x_2\end{array}}{\alpha\delta(1+\beta\delta-\delta)}$, when these four situations happen simultaneously, then $x_1 < \frac{\begin{array}{c}-2(1-\beta\delta)(2\alpha+\beta\delta)-3\alpha\beta\delta \\ +4c_1(1+\alpha-\beta\delta)+2c_2(1+\alpha-\beta\delta) \\ -2\beta x_2(1+\alpha-\beta\delta)\end{array}}{\alpha(4-3\beta\delta)}$. Therefore, $p_2^{NN*} > p_2^{DN*}$.

$$p_2^{NN*} - p_2^{ND*} = \frac{\beta\delta(\alpha-\beta\delta)+\beta\delta c_1+2\alpha c_2-\alpha\beta\delta x_1+\beta(2\alpha-\beta\delta)x_2}{4\alpha-\beta\delta} - \frac{\begin{array}{c}\alpha\delta(\alpha-\delta)+\delta(\alpha+\beta\delta-\delta)c_1+2\alpha(\alpha+\beta\delta-\delta)c_2 \\ -\alpha\delta(\alpha+\beta\delta-\delta)x_1+\alpha(2\alpha+\beta\delta-2\delta)x_2\end{array}}{\alpha(4\alpha+3\beta\delta-4\delta)}.$$

When $x_1 > \frac{\begin{array}{l}4\alpha\delta(\alpha-\delta)(\alpha-\beta\delta)+3\beta\delta\alpha\delta(\alpha-\delta+\beta\delta)\\+4\alpha\delta c_1(\alpha+\beta\delta-\delta)-\beta\delta\delta c_1(\alpha-\delta)+2\alpha\beta\delta\delta c_2\\+2\alpha x_2(2\alpha+\beta\delta-2\delta)(2\alpha-\beta\delta)\\+\alpha\beta\delta x_2[2\alpha-\delta-\delta(1+\beta)]\end{array}}{\alpha\delta[\alpha(4\alpha+3\beta\delta-4\delta)+\beta\delta\delta]}$, then $p_2^{NN*} > p_2^{ND*}$; otherwise $p_2^{NN*} < p_2^{ND*}$.

Since $\frac{\begin{array}{l}4\alpha\delta(\alpha-\delta)(\alpha-\beta\delta)+3\beta\delta\alpha\delta(\alpha-\delta+\beta\delta)\\+4\alpha\delta c_1(\alpha+\beta\delta-\delta)-\beta\delta\delta c_1(\alpha-\delta)+2\alpha\beta\delta\delta c_2\\+2\alpha x_2(2\alpha+\beta\delta-2\delta)(2\alpha-\beta\delta)\\+\alpha\beta\delta x_2[2\alpha-\delta-\delta(1+\beta)]\end{array}}{\alpha\delta[\alpha(4\alpha+3\beta\delta-4\delta)+\beta\delta\delta]} > \frac{\begin{array}{l}\delta(1-\delta)(2+2\alpha\delta-2\delta-\alpha+2\beta\delta-2\alpha\beta\delta)\\+\alpha\delta(1+\beta\delta-\delta)c_1\\-c_2[2(1+\alpha\delta-\delta)(1+\beta\delta-\delta)-\alpha\beta\delta]\\+[2(1+\alpha\delta-\delta)(1+\beta\delta-\delta)-\alpha\beta\delta]x_2\end{array}}{\alpha\delta(1+\beta\delta-\delta)}$,

when these four situations happen simultaneously,

then $x_1 < \frac{\begin{array}{l}4\alpha\delta(\alpha-\delta)(\alpha-\beta\delta)+3\beta\delta\alpha\delta(\alpha-\delta+\beta\delta)\\+4\alpha\delta c_1(\alpha+\beta\delta-\delta)-\beta\delta\delta c_1(\alpha-\delta)+2\alpha\beta\delta\delta c_2\\+2\alpha x_2(2\alpha+\beta\delta-2\delta)(2\alpha-\beta\delta)\\+\alpha\beta\delta x_2[2\alpha-\delta-\delta(1+\beta)]\end{array}}{\alpha\delta[\alpha(4\alpha+3\beta\delta-4\delta)+\beta\delta\delta]}$, $p_2^{NN*} < p_2^{ND*}$.

$$p_2^{ND*} - p_2^{DD*} = \frac{\begin{array}{l}\alpha\delta(\alpha-\delta)+\delta(\alpha+\beta\delta-\delta)c_1+2\alpha(\alpha+\beta\delta-\delta)c_2\\-\alpha\delta(\alpha+\beta\delta-\delta)x_1+\alpha(2\alpha+\beta\delta-2\delta)x_2\end{array}}{\alpha(4\alpha+3\beta\delta-4\delta)} - \frac{\begin{array}{l}\delta(1-\delta)(2+2\beta\delta-2\delta-\alpha-2\alpha\beta\delta+2\alpha\delta)\\+\alpha\delta(1+\beta\delta-\delta)c_1+2(1+\alpha\delta-\delta)(1+\beta\delta-\delta)c_2\\-\alpha\delta(1+\beta\delta-\delta)x_1+[2(1+\alpha\delta-\delta)(1+\beta\delta-\delta)-\alpha\beta\delta]x_2\end{array}}{4(1+\alpha\delta-\delta)(1+\beta\delta-\delta)-\alpha\beta\delta}.$$

When $x_1 < \frac{\begin{array}{l}\alpha\delta(\alpha-\delta)[4(1+\alpha\delta-\delta)(1+\beta\delta-\delta)-\alpha\beta\delta]\\-\delta\alpha(4\alpha+3\beta\delta-4\delta)(1-\delta)(2+2\beta\delta-2\delta-\alpha-2\alpha\beta\delta+2\alpha\delta)\\+\delta c_1(\alpha+\beta\delta-\delta)[4(1+\alpha\delta-\delta)(1+\beta\delta-\delta)-\alpha\beta\delta]\\-\alpha\delta\alpha c_1(4\alpha+3\beta\delta-4\delta)(1+\beta\delta-\delta)\\+2\alpha(\alpha+\beta\delta-\delta)c_2[4(1+\alpha\delta-\delta)(1+\beta\delta-\delta)-\alpha\beta\delta]\\-2\alpha c_2(4\alpha+3\beta\delta-4\delta)(1+\alpha\delta-\delta)(1+\beta\delta-\delta)\\+\alpha(2\alpha+\beta\delta-2\delta)x_2[4(1+\alpha\delta-\delta)(1+\beta\delta-\delta)-\alpha\beta\delta]\\-\alpha(4\alpha+3\beta\delta-4\delta)[2(1+\alpha\delta-\delta)(1+\beta\delta-\delta)-\alpha\beta\delta]x_2\end{array}}{\alpha\delta(1-\alpha)[4(1+\beta\delta-\delta)(\alpha+\beta\delta-\delta)(1-\delta)+\alpha\beta\delta]}$, then $p_2^{ND*} > p_2^{DD*}$;

otherwise $p_2^{ND*} < p_2^{DD*}$.

Since $\dfrac{\begin{gathered}\alpha\delta(\alpha-\delta)[4(1+\alpha\delta-\delta)(1+\beta\delta-\delta)-\alpha\beta\delta] \\ -\delta\alpha(4\alpha+3\beta\delta-4\delta)(1-\delta)(2+2\beta\delta-2\delta-\alpha-2\alpha\beta\delta+2\alpha\delta) \\ +\delta c_1(\alpha+\beta\delta-\delta)[4(1+\alpha\delta-\delta)(1+\beta\delta-\delta)-\alpha\beta\delta] \\ -\alpha\delta\alpha c_1(4\alpha+3\beta\delta-4\delta)(1+\beta\delta-\delta) \\ +2\alpha(\alpha+\beta\delta-\delta)c_2[4(1+\alpha\delta-\delta)(1+\beta\delta-\delta)-\alpha\beta\delta] \\ -2\alpha c_2(4\alpha+3\beta\delta-4\delta)(1+\alpha\delta-\delta)(1+\beta\delta-\delta) \\ +\alpha(2\alpha+\beta\delta-2\delta)x_2[4(1+\alpha\delta-\delta)(1+\beta\delta-\delta)-\alpha\beta\delta] \\ -\alpha(4\alpha+3\beta\delta-4\delta)[2(1+\alpha\delta-\delta)(1+\beta\delta-\delta)-\alpha\beta\delta]x_2\end{gathered}}{\alpha\delta(1-\alpha)[4(1+\beta\delta-\delta)(\alpha+\beta\delta-\delta)(1-\delta)+\alpha\beta\delta]} > \dfrac{\begin{gathered}\delta(1-\delta)(2+2\alpha\delta-2\delta-\alpha+2\beta\delta-2\alpha\beta\delta) \\ +\alpha\delta(1+\beta\delta-\delta)c_1 \\ -c_2[2(1+\alpha\delta-\delta)(1+\beta\delta-\delta)-\alpha\beta\delta] \\ +[2(1+\alpha\delta-\delta)(1+\beta\delta-\delta)-\alpha\beta\delta]x_2\end{gathered}}{\alpha\delta(1+\beta\delta-\delta)}$, when these four situations happen simultaneously,

then $x_1 < \dfrac{\begin{gathered}\alpha\delta(\alpha-\delta)[4(1+\alpha\delta-\delta)(1+\beta\delta-\delta)-\alpha\beta\delta] \\ -\delta\alpha(4\alpha+3\beta\delta-4\delta)(1-\delta)(2+2\beta\delta-2\delta-\alpha-2\alpha\beta\delta+2\alpha\delta) \\ +\delta c_1(\alpha+\beta\delta-\delta)[4(1+\alpha\delta-\delta)(1+\beta\delta-\delta)-\alpha\beta\delta] \\ -\alpha\delta\alpha c_1(4\alpha+3\beta\delta-4\delta)(1+\beta\delta-\delta) \\ +2\alpha(\alpha+\beta\delta-\delta)c_2[4(1+\alpha\delta-\delta)(1+\beta\delta-\delta)-\alpha\beta\delta] \\ -2\alpha c_2(4\alpha+3\beta\delta-4\delta)(1+\alpha\delta-\delta)(1+\beta\delta-\delta) \\ +\alpha(2\alpha+\beta\delta-2\delta)x_2[4(1+\alpha\delta-\delta)(1+\beta\delta-\delta)-\alpha\beta\delta] \\ -\alpha(4\alpha+3\beta\delta-4\delta)[2(1+\alpha\delta-\delta)(1+\beta\delta-\delta)-\alpha\beta\delta]x_2\end{gathered}}{\alpha\delta(1-\alpha)[4(1+\beta\delta-\delta)(\alpha+\beta\delta-\delta)(1-\delta)+\alpha\beta\delta]}$. Therefore, $p_2^{ND*} > p_2^{DD*}$.

$$p_2^{NN*} - p_2^{DD*} = \frac{\beta\delta(\alpha-\beta\delta)+\beta\delta c_1+2\alpha c_2-\alpha\beta\delta x_1+\beta(2\alpha-\beta\delta)x_2}{4\alpha-\beta\delta} - \frac{\begin{gathered}\delta(1-\delta)(2+2\beta\delta-2\delta-\alpha-2\alpha\beta\delta+2\alpha\delta) \\ +\alpha\delta(1+\beta\delta-\delta)c_1+2(1+\alpha\delta-\delta)(1+\beta\delta-\delta)c_2 \\ -\alpha\delta(1+\beta\delta-\delta)x_1+[2(1+\alpha\delta-\delta)(1+\beta\delta-\delta)-\alpha\beta\delta]x_2\end{gathered}}{4(1+\alpha\delta-\delta)(1+\beta\delta-\delta)-\alpha\beta\delta}.$$

When $x_1 < \dfrac{\begin{gathered}\beta\delta(\alpha-\beta\delta)[4(1+\alpha\delta-\delta)(1+\beta\delta-\delta)-\alpha\beta\delta] \\ -\delta(1-\delta)(4\alpha-\beta\delta)(2+2\beta\delta-2\delta-\alpha-2\alpha\beta\delta+2\alpha\delta) \\ +\beta\delta c_1[4(1+\alpha\delta-\delta)(1+\beta\delta-\delta)-\alpha\beta\delta] \\ -\alpha\delta c_1(1+\beta\delta-\delta)(4\alpha-\beta\delta) \\ +2\alpha c_2[4(1+\alpha\delta-\delta)(1+\beta\delta-\delta)-\alpha\beta\delta] \\ -2c_2(1+\alpha\delta-\delta)(1+\beta\delta-\delta)(4\alpha-\beta\delta) \\ +\beta x_2(2\alpha-\beta\delta)[4(1+\alpha\delta-\delta)(1+\beta\delta-\delta)-\alpha\beta\delta] \\ -x_2(4\alpha-\beta\delta)[2(1+\alpha\delta-\delta)(1+\beta\delta-\delta)-\alpha\beta\delta]\end{gathered}}{\alpha\delta[(1+\beta\delta-\delta)(4\beta+4\beta\alpha\delta-4\beta\delta-4\alpha+\beta\delta)-\alpha\beta\beta\delta]}$, then $p_2^{NN*} > p_2^{DD*}$; otherwise $p_2^{NN*} < p_2^{DD*}$.

Since
$$\frac{\begin{array}{l}\beta\delta(\alpha-\beta\delta)[4(1+\alpha\delta-\delta)(1+\beta\delta-\delta)-\alpha\beta\delta]\\ -\delta(1-\delta)(4\alpha-\beta\delta)(2+2\beta\delta-2\delta-\alpha-2\alpha\beta\delta+2\alpha\delta)\\ +\beta\delta c_1[4(1+\alpha\delta-\delta)(1+\beta\delta-\delta)-\alpha\beta\delta]\\ -\alpha\delta c_1(1+\beta\delta-\delta)(4\alpha-\beta\delta)\\ +2\alpha c_2[4(1+\alpha\delta-\delta)(1+\beta\delta-\delta)-\alpha\beta\delta]\\ -2c_2(1+\alpha\delta-\delta)(1+\beta\delta-\delta)(4\alpha-\beta\delta)\\ +\beta x_2(2\alpha-\beta\delta)[4(1+\alpha\delta-\delta)(1+\beta\delta-\delta)-\alpha\beta\delta]\\ -x_2(4\alpha-\beta\delta)[2(1+\alpha\delta-\delta)(1+\beta\delta-\delta)-\alpha\beta\delta]\end{array}}{\alpha\delta[(1+\beta\delta-\delta)(4\beta+4\beta\alpha\delta-4\beta\delta-4\alpha+\beta\delta)-\alpha\beta\beta\delta]}<\frac{\begin{array}{l}c_1[2(1+\alpha\delta-\delta)(1+\beta\delta-\delta)-\alpha\beta\delta]\\ -(1-\delta)(2+2\alpha\delta-2\delta+\beta\delta-\alpha\beta\delta)\\ -\beta(1+\alpha\delta-\delta)c_2+\beta(1+\alpha\delta-\delta)x_2\end{array}}{[2(1+\alpha\delta-\delta)(1+\beta\delta-\delta)-\alpha\beta\delta]},$$

when these four situations happen simultaneously,

then
$$x_1>\frac{\begin{array}{l}\beta\delta(\alpha-\beta\delta)[4(1+\alpha\delta-\delta)(1+\beta\delta-\delta)-\alpha\beta\delta]\\ -\delta(1-\delta)(4\alpha-\beta\delta)(2+2\beta\delta-2\delta-\alpha-2\alpha\beta\delta+2\alpha\delta)\\ +\beta\delta c_1[4(1+\alpha\delta-\delta)(1+\beta\delta-\delta)-\alpha\beta\delta]\\ -\alpha\delta c_1(1+\beta\delta-\delta)(4\alpha-\beta\delta)\\ +2\alpha c_2[4(1+\alpha\delta-\delta)(1+\beta\delta-\delta)-\alpha\beta\delta]\\ -2c_2(1+\alpha\delta-\delta)(1+\beta\delta-\delta)(4\alpha-\beta\delta)\\ +\beta x_2(2\alpha-\beta\delta)[4(1+\alpha\delta-\delta)(1+\beta\delta-\delta)-\alpha\beta\delta]\\ -x_2(4\alpha-\beta\delta)[2(1+\alpha\delta-\delta)(1+\beta\delta-\delta)-\alpha\beta\delta]\end{array}}{\alpha\delta[(1+\beta\delta-\delta)(4\beta+4\beta\alpha\delta-4\beta\delta-4\alpha+\beta\delta)-\alpha\beta\beta\delta]}.$$
Therefore, $p_2^{NN*}<p_2^{DD*}$.

Proof of Proposition 6.

$$\pi_{M_1}^{NN*}-\pi_{M_1}^{DN*}=\frac{[2\alpha(\alpha-\beta\delta)-(2\alpha-\beta\delta)c_1+\alpha c_2+\alpha(2\alpha-\beta\delta)x_1-\alpha\beta x_2]^2}{(\alpha-\beta\delta)(4\alpha-\beta\delta)^2}$$

（1） $$-\alpha\frac{\begin{array}{l}[(1-\beta\delta)(2+\alpha\beta\delta-\beta\delta)-(2+\alpha\beta\delta-2\beta\delta)c_1+(1+\alpha\beta\delta-\beta\delta)c_2\\ +(2+\alpha\beta\delta-2\beta\delta)x_1-\beta(1+\alpha\beta\delta-\beta\delta)x_2]^2\end{array}}{(1-\beta\delta)(4+3\alpha\beta\delta-4\beta\delta)^2}+C_{d1}.$$

When
$$C_{d1}<\alpha\frac{\begin{array}{l}[(1-\beta\delta)(2+\alpha\beta\delta-\beta\delta)-(2+\alpha\beta\delta-2\beta\delta)c_1+(1+\alpha\beta\delta-\beta\delta)c_2\\ +(2+\alpha\beta\delta-2\beta\delta)x_1-\beta(1+\alpha\beta\delta-\beta\delta)x_2]^2\end{array}}{(1-\beta\delta)(4+3\alpha\beta\delta-4\beta\delta)^2}-\frac{[2\alpha(\alpha-\beta\delta)-(2\alpha-\beta\delta)c_1+\alpha c_2+\alpha(2\alpha-\beta\delta)x_1-\alpha\beta x_2]^2}{(\alpha-\beta\delta)(4\alpha-\beta\delta)^2},$$
then $\pi_{M_1}^{NN*}<\pi_{M_1}^{DN*}$;

otherwise, $\pi_{M_1}^{NN*}>\pi_{M_1}^{DN*}$.

(2) $$\pi_{M_1}^{ND*}-\pi_{M_1}^{DD*}=\frac{\begin{array}{c}(\alpha+\beta\delta-\delta)[2\alpha(\alpha-\delta)-(2\alpha+\beta\delta-2\delta)c_1+\alpha\beta c_2\\+\alpha(2\alpha+\beta\delta-2\delta)x_1-\alpha\beta x_2]^2\end{array}}{\alpha(\alpha-\delta)(4\alpha+3\beta\delta-4\delta)^2}-\alpha(1+\beta\delta-\delta)\frac{\begin{array}{c}\{(1-\delta)(2+2\alpha\delta-2\delta+\beta\delta-\alpha\beta\delta)\\-[2(1+\alpha\delta-\delta)(1+\beta\delta-\delta)-\alpha\beta\delta]c_1+\beta(1+\alpha\delta-\delta)c_2\\+[2(1+\alpha\delta-\delta)(1+\beta\delta-\delta)-\alpha\beta\delta]x_1-\beta(1+\alpha\delta-\delta)x_2\}^2\end{array}}{(1-\delta)[4(1+\alpha\delta-\delta)(1+\beta\delta-\delta)-\alpha\beta\delta]^2}+C_{d1}.$$

When $C_{d1}<\alpha(1+\beta\delta-\delta)\frac{\begin{array}{c}\{(1-\delta)(2+2\alpha\delta-2\delta+\beta\delta-\alpha\beta\delta)\\-[2(1+\alpha\delta-\delta)(1+\beta\delta-\delta)-\alpha\beta\delta]c_1+\beta(1+\alpha\delta-\delta)c_2\\+[2(1+\alpha\delta-\delta)(1+\beta\delta-\delta)-\alpha\beta\delta]x_1-\beta(1+\alpha\delta-\delta)x_2\}^2\end{array}}{(1-\delta)[4(1+\alpha\delta-\delta)(1+\beta\delta-\delta)-\alpha\beta\delta]^2}-\frac{\begin{array}{c}(\alpha+\beta\delta-\delta)[2\alpha(\alpha-\delta)-(2\alpha+\beta\delta-2\delta)c_1+\alpha\beta c_2\\+\alpha(2\alpha+\beta\delta-2\delta)x_1-\alpha\beta x_2]^2\end{array}}{\alpha(\alpha-\delta)(4\alpha+3\beta\delta-4\delta)^2}$, then $\pi_{M_1}^{ND*}<\pi_{M_1}^{DD*}$; otherwise, $\pi_{M_1}^{ND*}>\pi_{M_1}^{DD*}$.

Proof of Proposition 7.

(1) $$\pi_{M_2}^{NN*}-\pi_{M_2}^{ND*}=\alpha\frac{\begin{array}{c}[\beta\delta(\alpha-\beta\delta)+\beta\delta c_1-(2\alpha-\beta\delta)c_2\\-\alpha\beta\delta x_1+\beta(2\alpha-\beta\delta)x_2]^2\end{array}}{\beta\delta(\alpha-\beta\delta)(4\alpha-\beta\delta)^2}-\frac{\begin{array}{c}\beta[\alpha\delta(\alpha-\delta)+\delta(\alpha+\beta\delta-\delta)c_1-\alpha(2\alpha+\beta\delta-2\delta)c_2\\-\alpha\delta(\alpha+\beta\delta-\delta)x_1+\alpha(2\alpha+\beta\delta-2\delta)x_2]^2\end{array}}{\alpha\delta(\alpha-\delta)(4\alpha+3\beta\delta-4\delta)^2}+C_{d2}.$$

When $C_{d2}<\frac{\begin{array}{c}\beta[\alpha\delta(\alpha-\delta)+\delta(\alpha+\beta\delta-\delta)c_1-\alpha(2\alpha+\beta\delta-2\delta)c_2\\-\alpha\delta(\alpha+\beta\delta-\delta)x_1+\alpha(2\alpha+\beta\delta-2\delta)x_2]^2\end{array}}{\alpha\delta(\alpha-\delta)(4\alpha+3\beta\delta-4\delta)^2}-\alpha\frac{\begin{array}{c}[\beta\delta(\alpha-\beta\delta)+\beta\delta c_1-(2\alpha-\beta\delta)c_2\\-\alpha\beta\delta x_1+\beta(2\alpha-\beta\delta)x_2]^2\end{array}}{\beta\delta(\alpha-\beta\delta)(4\alpha-\beta\delta)^2}$, then $\pi_{M_2}^{NN*}<\pi_{M_2}^{ND*}$; otherwise, $\pi_{M_2}^{NN*}>\pi_{M_2}^{ND*}$.

(2) $$\pi_{M_2}^{DN*}-\pi_{M_2}^{DD*}=\frac{(1+\alpha\beta\delta-\beta\delta)[\beta\delta(2-\alpha)(1-\beta\delta)+\alpha\beta\delta c_1-(2+\alpha\beta\delta-2\beta\delta)c_2-\alpha\beta\delta x_1+\beta(2+\alpha\beta\delta-2\beta\delta)x_2]^2}{\beta\delta(1-\beta\delta)(4+3\alpha\beta\delta-4\beta\delta)^2}-\beta(1+\alpha\delta-\delta)\frac{\{\delta(1-\delta)(2+2\beta\delta-2\delta-\alpha-2\alpha\beta\delta+2\alpha\delta)+\alpha\delta(1+\beta\delta-\delta)c_1-[2(1+\alpha\delta-\delta)(1+\beta\delta-\delta)-\alpha\beta\delta]c_2-\alpha\delta(1+\beta\delta-\delta)x_1+[2(1+\alpha\delta-\delta)(1+\beta\delta-\delta)-\alpha\beta\delta]x_2\}^2}{\delta(1-\delta)[4(1+\alpha\delta-\delta)(1+\beta\delta-\delta)-\alpha\beta\delta]^2}+C_{d2}.$$

When $$C_{d2}<\beta(1+\alpha\delta-\delta)\frac{\{\delta(1-\delta)(2+2\beta\delta-2\delta-\alpha-2\alpha\beta\delta+2\alpha\delta)+\alpha\delta(1+\beta\delta-\delta)c_1-[2(1+\alpha\delta-\delta)(1+\beta\delta-\delta)-\alpha\beta\delta]c_2-\alpha\delta(1+\beta\delta-\delta)x_1+[2(1+\alpha\delta-\delta)(1+\beta\delta-\delta)-\alpha\beta\delta]x_2\}^2}{\delta(1-\delta)[4(1+\alpha\delta-\delta)(1+\beta\delta-\delta)-\alpha\beta\delta]^2}-\frac{(1+\alpha\beta\delta-\beta\delta)[\beta\delta(2-\alpha)(1-\beta\delta)+\alpha\beta\delta c_1-(2+\alpha\beta\delta-2\beta\delta)c_2-\alpha\beta\delta x_1+\beta(2+\alpha\beta\delta-2\beta\delta)x_2]^2}{\beta\delta(1-\beta\delta)(4+3\alpha\beta\delta-4\beta\delta)^2}$$, then

$\pi_{M_2}^{DN*}<\pi_{M_2}^{DD*}$; otherwise, $\pi_{M_2}^{DN*}>\pi_{M_2}^{DD*}$.
